# Biological functions of sialic acid as a component of bacterial endotoxin

**DOI:** 10.3389/fmicb.2022.1028796

**Published:** 2022-10-20

**Authors:** Bartłomiej Dudek, Jacek Rybka, Gabriela Bugla-Płoskońska, Agnieszka Korzeniowska-Kowal, Bożena Futoma-Kołoch, Aleksandra Pawlak, Andrzej Gamian

**Affiliations:** ^1^Department of Microbiology, University of Wrocław, Wrocław, Poland; ^2^Department of Immunology of Infectious Diseases, Hirszfeld Institute of Immunology and Experimental Therapy, Polish Academy of Sciences, Wrocław, Poland

**Keywords:** lipopolysaccharide, O-antigen, molecular mimicry, pathogenesis, outer membrane, Gram-negative bacteria, sialic acid

## Abstract

Lipopolysaccharide (endotoxin, LPS) is an important Gram-negative bacteria antigen. LPS of some bacteria contains sialic acid (Neu5Ac) as a component of O-antigen (O-Ag), in this review we present an overview of bacteria in which the presence of Neu5Ac has been confirmed in their outer envelope and the possible ways that bacteria can acquire Neu5Ac. We explain the role of Neu5Ac in bacterial pathogenesis, and also involvement of Neu5Ac in bacterial evading the host innate immunity response and molecular mimicry phenomenon. We also highlight the role of sialic acid in the mechanism of bacterial resistance to action of serum complement. Despite a number of studies on involvement of Neu5Ac in bacterial pathogenesis many aspects of this phenomenon are still not understood.

## Introduction

Lipopolysaccharide (endotoxin, LPS) is a component of the outer membrane (OM) of Gram-negative bacterial cell, facing the external environment. The unique chemical structure of LPS give bacterial cells a number of specific properties: LPS constitutes a strong barrier around the cell, giving higher resistance to toxic chemicals, such as antibiotics or detergents. It also enables the survival in hostile environments during host colonization or infection (Gunn, 2000), such as the action of the complement and phagocytic cells’ lytic activity of the host immune system (Bravo et al., 2008; Dudek et al., 2016). The LPS influences the host organism particularly after the lysis of the bacterial cell. Consequently, the released LPS may trigger the development of septic shock (Ramachandran, 2014).

**Figure 1:**
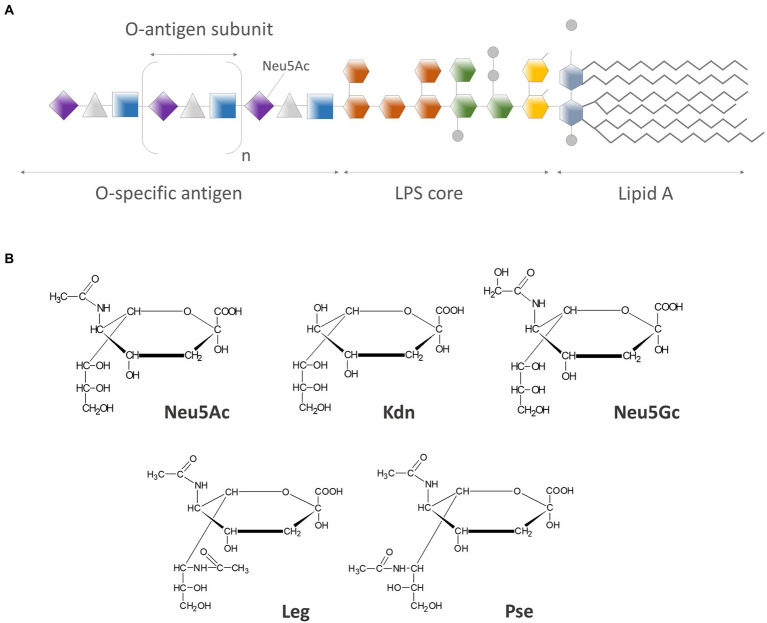
**(A)** Schematic structure of LPS molecule: O-specific antigen chain has a variable length, thus the amount of sialic acid residues per LPS molecule can vary from none (in rough LPS) up to over one hundred (in so-called very-long-O-antigen LPS); **(B)** Chemical structures of most common members of neuraminic acids family: N-acetylneuraminic acid (Neu5Ac), 2-keto-3-deoxy-nonulosonic acid (Kdn), N-glycolylneuraminic acid (Neu5Gc), legionaminic acid (Leg), and pseudaminic acid (Pse), presented in simplified Fisher projection formula.

LPS consists of three structural domains: lipid A, oligosaccharide core, and O-antigen (O-Ag). O-Ag is composed of oligosaccharide building blocks called repeating units. The number of these units in O-Ag can vary: from one up to over one hundred even in one strain (Rhee, 2014). The LPS with O-antigen is called smooth LPS in contrast to the rough LPS that lacks O-Ag. In mucosal pathogens often can be found the structure called lipooligosaccharide (LOS), which is the truncated version of LPS, where one or more small oligosaccharide branches are attached directly to the inner core connected with Lipid A (Raetz and Whitfield, 2002). The O-Ag, having high structural diversity among bacterial strains, is a distal part of LPS and contributes to its immunogenic properties (Figure 1A; Feng et al., 2004).

Sialic acids (Sias) are a group of 9-carbon α-keto acidic monosaccharides related to nonulosonic acid, characterized by the great level of structural diversity. They are usually localized at the non-reducing end of oligosaccharide chains on glycoproteins and glycolipids (Tiralongo, 2010). The family of sialic acids consists of more than 80 members, with the *N-*acetylneuraminic acid (Neu5Ac) being the most prevalent member of this large group (Figure 1B; Varki and Schauer, 2009; Lewis et al., 2016). They are essential components of glycoproteins and glycoconjugates (Varki and Schauer, 2009). Neu5Ac has been generally found in vertebrates, but not in plants nor in invertebrates or most prokaryotes. It can however constitute a component of some bacterial surface structures like capsular polysaccharides, lipopolysaccharides, or oligosaccharides (Traving and Schauer, 1998). Sialic acids have high level of structural variability due to an array of possible substituents (like amino, glycolyl, or acetyl groups) on the carbohydrate backbone (Varki, 1992), which is the basis of their diverse biological functions (Schauer, 2009).

In macromolecules Sias are connected by a very labile ketosidic bond, what is the result of sialic acid structure: the 3-deoxy moiety adjacent to the ketosidic group at C2. The studies on polysaccharides containing Neu5Ac require therefore the specific methodology to avoid the degradation of the polysaccharide chain and loss or migration of O-acetyl substituents (Schauer and Kamerling, 2018). Sias are usually glycosidically linked to galactose, *N-*acetylglucosamine, *N*-acetylgalactosamine or are found as α-2,8-linked homopolymers (Bhattacharjee et al., 1975; Tiralongo, 2010). There are two types of Sias diversity. The first one is a result of different α-linkages formed between the C-2 of Sias and underlying sugars. The second type of diversity is the effect of a variety of natural modifications, including mainly the substitution of O-acetyl group at positions C4, C7, C8, or C9 of sialic acid molecule as well as the modification with N-glycolyl group (Varki and Schauer, 2009).

Generally, sialic acids contribute to multiple cellular mechanisms and play a significant role as ligands for various molecules, such as hormones, cytokines, antibodies, and also viruses and bacteria (Varki, 2008; Schauer, 2009). Sias are highly expressed on animal outer cell membranes. For example, there are more than 10 million molecules of Sias per human erythrocyte. Also, lysosomal membranes and secreted glycoproteins are rich in Sias. That is why the putative role of Sias is the stabilization of molecules and membranes and modulation of interactions with the cell environment (Varki and Schauer, 2009). Some other roles of Sias in animals include: binding and transport of ions and drugs, stabilizing the conformation of proteins, enhancing the viscosity of mucins, protection of molecules and cells from the action of proteases or glycosidases, modulation of processes involved in transmembrane signaling, growth, and fertilization. Sias are also ligands for a variety of microbial and animal lectins.

## Sialic acids as components of bacterial cells

Sialic acid is rarely the component of the bacterial cell and it can be found only in some bacterial species. One of the first described structures containing sialic acid was α-2,8-*N-*acetylneuraminic acid polymer, so-called colominic acid, which was found in the capsule of the *Escherichia coli* K1 (Barry, 1958; Bolaños and DeWitt, 1966) and can be further modified by *O-*acetyl groups (Yang et al., 2021). Sialic acid may also be present as a component of other capsular polysaccharides: e.g. *E. coli* K92, *E. coli* K9, *Neisseria meningitidis* groups B and C and *Streptococcus agalactiae* group B (Masson et al., 1982; Severi et al., 2005; Khatua et al., 2010) or several *Streptococcus suis* serotypes (Van Calsteren et al., 2013). The formation of polysialic acid capsules is an important factor of the pathogenicity and survival of those bacteria in host organisms (Ringenberg et al., 2003). Sialylation of the structures on the cell surface has an impact on the adherence of bacteria to host cells receptors (Siglecs—Sialic acid-binding immunoglobulin-type lectins), formation of the bacterial biofilm and resistance to bactericidal action of serum (Mielnik et al., 2001; Feng et al., 2012; Spinola et al., 2012; Huizinga et al., 2013). For years, researchers suggested the possibility that sialic acid is a component of LPS (Barry, 1959; Dewitt and Rowe, 1961), these assumptions have been confirmed by showing the presence of sialic acid in the O-Ag of LPS of *Hafnia alvei* 2 (PCM 2386, Polish Collection of Microorganisms; Gamian et al., 1991).

**Figure 2:**
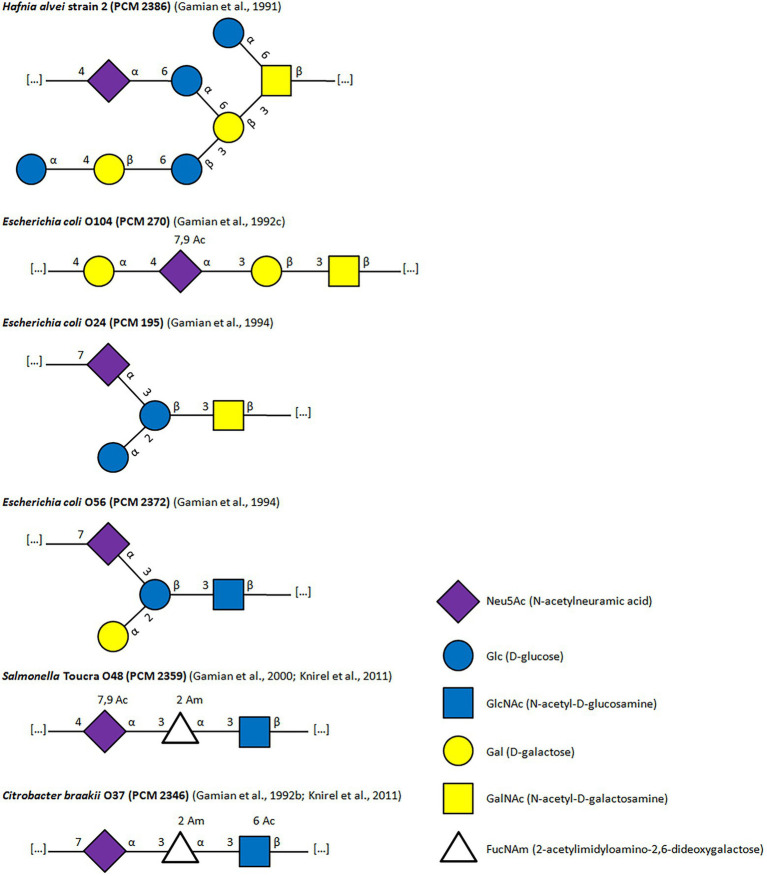
Structures of LPS containing Neu5Ac as a component of O-Ag.

By now Neu5Ac has been found in LPS of other strains like *E. coli* O24 (PCM 195), O56 (PCM 2372), O104 (PCM 270), O171, *Citrobacter braakii* O37 (PCM 2346), *Pseudomonas aeruginosa, Salmonella* Toucra O48 (PCM 2359), *Fusobacterium nucleatum,* or *Rhodobacter capsulatus* and in LOS of strains like *Campylobacter jejuni* O1, *C. jejuni* O4, *C. jejuni* O23, *Vibrio, Neisseria,* or *Haemophilus influenzae* (Gamian et al., 1991, 1992b,c, 1994, 2000 and references cited herein, Mielnik et al., 2001 and citations herein, Krauss et al., 1992; Ali et al., 2006; Vinogradov et al., 2017; Schauer and Kamerling, 2018). Gamian et al. have described the structures of six bacterial polysaccharides (LPS fragments), containing sialic acid, namely O-specific antigens of *C. braakii* O37, *H. alvei* PCM2386, *E. coli* O24, O56, O104, and *S.* Toucra O48 (Figure 2). In these studied strains, the sialic acid residues are located within the of O-specific polysaccharide chain, in all of its oligosaccharide subunits. Each LPS molecule has the sialic residue at the non-reducing end, except for *E. coli* O104, where the terminal sugar is the galactose residue (Gamian and Kenne, 1993). Internal sialic acid is substituted at the C-4 position in polysaccharide chains of *E. coli* O104 (Gamian et al., 1992c), *H. alvei,* and *S.* Toucra O48 (Gamian et al., 2000) by Gal, GalNAc, GlcNAc, respectively, while in *E. coli* O24, O56 and *C. braakii* O37 a new type of glycosylation at position C7 of Neu5Ac with GalNAc, GlcNAc, and 6-OAc-GlcNAc, respectively, have been found (Figure 2; Gamian and Kenne, 1993; Gamian et al., 1994). The epitopes recognized by the antibodies reside within the polysaccharide chain and include sialic acid along with neighboring sugar residues in almost all antigens tested. The exception is the *H. alvei* 2 antigen, where the epitope is located in a large, branched, octasaccharide subunit without the participation of Sias (Gamian et al., 1991).

New methods allow for a high throughput screening of various bacterial environments for sialic acid. The analysis of human microbiome showed new *E. coli* strain carrying Neu5Ac on its surface (Han et al., 2021). Recent findings give an insight into the presence of sialic acid-producing bacteria in unexpected sources: analysis of anionic polymers of anaerobic ammonium oxidation bacteria *Candidatus Brocadia sapporoensis* shows the sialic acid presence in the extracellular polymeric substances (Boleij et al., 2020), genomic and biochemical analysis indicates the production of sialic acid in bacteria *Candidatus Accumulibacter*, one bacteria found in wastewater treatment plants (de Graaff et al., 2019). The findings suggest that the sialic acid role is even broader in the context of environmental nonpathogenic bacteria than previously thought.

## The role of sialic acids in the pathogenesis of bacteria

Microorganisms can use sialic acid in a strategy that helps them to thrive in the host. The strategy, called molecular mimicry, occurs when antigenic determinants of pathogens are similar to host molecules, which can induce an autoimmunological response. Sialic acids, widely distributed in vertebrates, mediate and/or modulate many physiological and pathological processes. Neu5Ac which is displayed on extended glycan chains in microbe’s envelopes in some cases almost perfectly mimic mammalian structures. Pathogens covered with sialic acid molecules can circumvent, subvert or even inhibit host innate immunity function (Gowthaman and Eswarakumar, 2013). Sias presented on bacterial cells can mask the antigenic sites, what allows bacteria to avoid the immune response of the host (Moran et al., 2009; Tiralongo, 2010). Neu5Ac present in pathogen structures like LPS, LOS or capsular polysaccharide leads to lower immunogenicity of host through:

- controlling of the alternative complement pathway by binding Neu5Ac to complement factor H;
- change the TLR4-mediated response to the pathogen;
- sending a negative signal to innate immune cells *via* CD33-related Siglecs (Varki, 2008, 2017).

Some mucosal pathogens, like *N. meningitidis*, *H. influenzae,* or *C. jejuni* have LOS replacing LPS in their outer membrane. Despite the lack of O-Ag, the phase variation is realized by the differential expression of the oligosaccharides attached to the inner core, therefore the bacteria still can present the diversity of structural epitopes (Raetz and Whitfield, 2002). *Neisseria meningitidis* group B that reside in the nasopharyngeal mucosa of healthy people, in some cases can be a cause of meningococcal meningitis. These bacteria carries polysialic acid envelope, that protects its cells against the bactericidal effect of the serum (Nicholson and Lepow, 1979). An additional protection from a host immune response may be provided by the LOS containing sialic acid residues. Most isolated pathogens causing meningococcal meningitis are strains of immunotype L3,7,9 with the terminal structure of the lacto-*N*-neo-tetraose unit (LNnT) in the LOS molecule (Jennings et al., 1983). Presence of LNnT unit was also found in lipopolysaccharides of L2 immunotype (Gamian et al., 1992a) and L5 (Michon et al., 1990). LNnT unit is undistinguishable from the structure present on the paragloboside precursor of the glycolipid antigen on human erythrocytes ABH (Hakomori, 1981) and on the surface antigen of lymphocytes and granulocytes (Spitalnik et al., 1985). The lacto-*N*-neo-tetraose moiety is also present on human milk oligosaccharides (Yamashita et al., 1976). The galactose of the lacto-*N*-neo-tetraose (LNnT) residue action can be substituted with sialic acid by α-2,3-sialyl transferase (Mandrell et al., 1991), and then this structure resembles human sialylparaglobosides. LNnT residues can be found also in the LOS structures of *Neisseria gonorrhoeae* (John et al., 1999). This bacterial species causes purulent urogenital infections that can lead to infertility if left untreated. In *N. gonorrhoeae* lipopolysaccharide *N-*acetyl-D-lactosamine (LacNAc) is present and its α-2,3-substituting sialic acid mimics sugar moiety of human cells glycoproteins, such as the erythrocyte surface protein Band 3. Individual feature of the species *N. gonorrhoeae* is the ability to sialylation of LOS by gonococcal α-2,3-sialyltransferase using the activated form of sialic acid (CMP-Neu5Ac) present in human body fluids (Mandrell et al., 1990).

*Haemophilus ducreyi* causes a sexually transmitted disease that is common in the population of developing countries in Africa, Southeast Asia and South America (Jessamine and Ronald, 1990). LOS, in addition to the cytotoxins produced by *H. ducreyi*, is an important virulence factor, and its properties enable the adherence of bacterial cells to fibroblasts and human endothelial keratinocytes, what greatly facilitate the penetration of the host tissues by bacteria. The LOS biosynthesis occurs in two ways: through α-2,3-substitution with the sialic acid residue (85%), to give sialyl-*N-*acetyllactosamine, or substitution of another *N-*acetyllactosamine residue to the terminal galactose (Schweda et al., 1995). The resulting structures resemble paraglobosides and other glycosphingolipids of human tissues.

Studies on the possible contribution of LPS sialic acid to the bacterial pathogenicity have shown that rabbit serum anti-*C. braakii* O37 agglutinates human and equine erythrocytes. Gamian et al. have shown that the sugar structures recognized on the Band 3 protein of the red blood cells are responsible for the agglutination of human erythrocytes by these antibodies. In addition, preliminary histological tests indicate that anti-*C. braakii* O37 antibodies also interact with human brain tissue. Such cross-reactivity suggests the presence of common bacterial and host epitopes, which is a good example of mimicking the host structures by bacteria. Consequently, the bacterial cells of *C. braakii* O37 are completely resistant to the bactericidal action of complement from human blood and also from bovine sera (Ebaid et al., 2008).

Recent studies showed that anti-O24 and anti-O56 *E. coli* antibodies have affinity to various human tissues. The presence of antigens on various tissues, cross-reacting with those antibodies, was tissue specific and O56 antibody performed better than O24 in staining epithelial and nervous tissues. Positive tissue staining was also observed for both normal ganglia and ganglioneuroma tumor tissues. The remarkable observation is that the cytoplasmic epitope recognized by anti-O56 antibodies is a new marker specific for glandular epithelium and nervous tissue (Korzeniowska-Kowal et al., 2015).

The phenomenon of molecular mimicry is connected with the occurrence of autoimmunity. The immunodeterminants of the microorganism and host can be sufficiently similar to trigger a cross-reactivity, but different enough to overcome immunological tolerance (Albert and Inman, 1999). In the autoimmunization process the autoantibodies are produced since bacterial antigens are similar or even identical to the host antigens (Mason, 1992). Guillain–Barré syndrome (GBS) is one of the best described examples of disease elicited by molecular mimicry (Hahn, 1998). GBS may occur as a complication after infection of the digestive tract caused by *Campylobacter* strains.

Sialylation of *C. jejuni* LOS is associated with some cases of the disease. The autoantibodies raised against that LOS recognize gangliosides of the peripheral nerves of the host due to the identical structure of both structures (Avril et al., 2006; Heikema et al., 2010; Chang and Nizet, 2014). It was observed that in almost 40% of patients with GBS, the appearance of nervous system symptoms was induced by an acute gastrointestinal infection caused by *C. jejuni* serostrains with LPS molecule (O:4, O:19, O:23, and O:36) or LOS molecule (O:1,O:2; Mishu and Blaser, 1993; Aspinall et al., 1994; Penner and Aspinall, 1997). Highly elevated concentrations of antibodies directed against various gangliosides constituting a component of peripheral myelin were found in these patients’ sera (Hao et al., 1998). The LPS structure of *C. jejuni* O19 is similar to four gangliosides GD3, GM1, GD1a, and GT1a (Nachamkin et al., 1999). A variation of Guillain–Barré disease is a condition called Miller Fisher syndrome (MFS) characterized by ocular paralysis (ophthalmoplegia), ataxia, and areflexia. These symptoms occur after infections mostly caused by *C. jejuni,* serotype O2, and to some extent serotype O10 (Yuki et al., 1997; Sandler et al., 2015). In patients with acute phase of the disease, high levels of IgG antibodies are directed against GQ1b gangliosides but also to lesser extent against GT1a were found (Jacobs et al., 1997). These antibodies cross-react with the whole *C. jejuni* cells and with the lipooligosaccharide fraction from the bacterial cell wall (Yuki et al., 1994). The treatment of bacterial cell or LOS with sialidase eliminates this antibodies binding. It demonstrates that sialic acid on the surface of the bacterial cell is a component of the cross-reacting epitope. Additionally bipolar flagella of *C. jejuni* are heavily O-glycosylated with microbial sialic acids, what additionally helps in evasion of host immunological response (Meng et al., 2021).

Studies on the structure and function of bacterial antigens, that mimic tissue epitopes, may have a great impact on effective and safe vaccines manufacture (Sheikhi Moghaddam et al., 2021). It can also explain the mechanisms of tolerance to pathogenic factors, overcoming this tolerance and inducing production of autoantibodies. In addition, such studies may allow to find structural elements that would be the target of novel drugs for the inhibition of the biosynthesis of bacterial polysaccharides or inhibition of the adhesion to host cells and colonization of its tissues. Some antigens, such as bacterial colominic acid, that also can be found in fetus development or in some types of cancers, is used by bacteria to evade human host cells and might be used in vaccines development (Sato and Kitajima, 2021) or serve as a model in the development of new oncotherapy (Korzeniowska-Kowal et al., 2001).

## The role of Neu5Ac in bacterial resistance to mechanisms of innate immunity of the host

The bacterial cells expressing sialylated lipopolysaccharide are much more resistant to the bactericidal effect of serum, e.g., there is no binding of the IgM to the bacterial surface, which results in the inhibition of the complement classical pathway (CP; Wetzler et al., 1992). Activation of the human complement pathways by bacterial cell-associated sialic acid has been well documented on the serogroup B strains of *N. meningitidis* (Jarvis and Vedros, 1987; Vogel et al., 1997; Unkmeir et al., 2002). For example, enzymatic desialylation of B16B6 strain, initially resistant to the AP-mediated bactericidal activity, resulted in an increase in the amount of C3 binding, accompanied also by an increase in factor B deposition (Jarvis and Vedros, 1987). Using *N. meningitidis* isogenic mutants, deficient in capsule expression or sialylation of the LOS (a *galE* mutant) or both (a mutant with a deletion of the *cps* gene locus), showed that complement factor C3b linkage was more pronounced in *galE* mutants with nonsialylated LOS than in meningococci with wild-type LOS, irrespective of the capsule phenotype. C3b deposition was caused by both: the classical and the alternative pathways (CP and AP), or only CP, depending of the serum concentration. Authors conclude that serum resistance requires capsule expression and can be partly explained by C3b linkage pattern (Vogel et al., 1997). A different view on meningococcal LOS was presented by Unkmeir et al. who analyzed the roles of meningococcal LOS and capsule expression in the interaction of *N. meningitidis* with human dendritic cells (DC). It turned out that serogroup B mutant strain lacking LOS expression barely led to cytokine induction, nevertheless sialylation of LOS did not influence cytokine secretion by DC. However, it was found that the phagocytosis of *N. meningitidis* by human DC is inhibited by LOS sialylation (Unkmeir et al., 2002).

The sialylated LOS of *H. influenzae* and *N. gonorrhoeae* inhibits the activation of the alternative pathway of the complement system (Ram et al., 1998; Figueira et al., 2007). The presence of sialylated LOS in *N. gonorrhoeae* increases the binding of factor H to the surface of the bacterial cell. Interestingly, the factor H binding to the cell requires the presence of gonococcal PorB protein, suggesting that LOS and porin may form a complex epitope or that LOS sialylation may cause a conformational change in LPS chains, revealing binding sites in the PorB protein (Madico et al., 2007). *C. jejuni* and *N. meningitidis* with sialylated LOS can interact with lectins specific for sialic acid: sialoadhesins are expressed mainly on the surface of hematopoetic system cells and belong to the Siglecs family, e.g., CD22 proteins on B cells and CD33 on monocytes and macrophages. Probably the ligand-bacteria interactions are the other way in which the sialylation on the surface of bacterial cells can modulate the immune response of the host (Jones et al., 2003; Avril et al., 2006; Carlin et al., 2007).

*Pseudomonas aeruginosa* is an opportunistic pathogen that colonizes immunocompromised individuals, including patients with cystic fibrosis or burn victims. It has been shown that *P. aeruginosa*, while devoid of *de novo* sialic acid synthesis, is able to uptake the sugar from the exogenous substrate rich in Neu5Ac. The acquired sialic acid showed a distinct ability to reduce the deposition of complement C3b on the surface of the bacterial cell, in addition, it presented high affinity for human CD33 receptors, including the Siglec receptors 3, 5, 7, 9, and 10 (Khatua et al., 2010; Chang and Nizet, 2014). Khatua et al. also showed that sialic acid acquired by *P. aeruginosa* may decrease neutrophil activation, what leads to the attenuation of the innate host response against the pathogen (Khatua et al., 2010). Additionally, sialylated *P. aeruginosa* suppress macrophage antimicrobial responses and inhibit phagosome maturation, thereby it remains persistently viable and replicates intracellularly in macrophages (Mukherjee et al., 2020).

**Figure 3:**
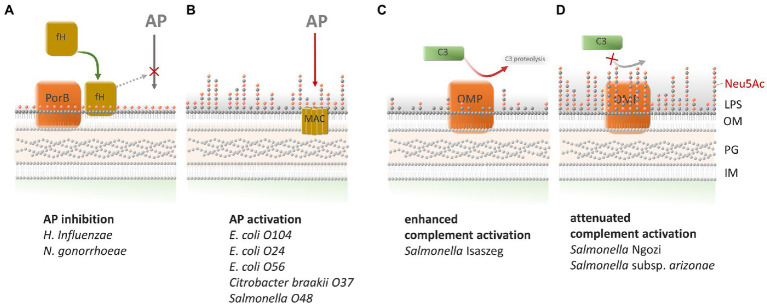
Correlation between the presence of NeuAc in bacterial LPSs and complement activation. There are four possible ways of C activation depending on sialylated LPSs and outer membrane proteins. **(A)** The sialylated LOS of *Haemophilus influenzae* and *Neisseria gonorrhoeae* inhibits the activation of the alternative pathway (AP) of the complement system by binding of factor H. The binding of factor H requires the presence of gonococcal outer membrane protein PorB (Ram et al., 1998; Figueira et al., 2007; Madico et al., 2007). **(B)** *Enterobacteriaceae* such as *Escherichia coli* O104, *E. coli* O24, *E. coli* O56, *Citrobacter braakii* O37 and *Salmonella* O48 were killed *via* the AP pathway activation although they possessed sialylated LPSs (Bugla-Płoskońska and Doroszkiewicz, 2006; Bugla-Płoskońska et al., 2010a,b). **(C)** LPS with a low content (near the limit of detection) of sialic acid in LPS of *Salmonella* Isaszeg enabled C3 activation on OMPs in the range of molecular masses of 35–48 kDa (Futoma-Kołoch et al., 2015). **(D)** LPS with a high content of sialic acid of *S.* Ngozi and *S.* subsp. *arizonae* impeded C3 activation by OMPs in the range of molecular masses of 35–48 kDa (Futoma-Kołoch et al., 2015).

Neu5Ac could be used as an indicator of bacteria resistance to bactericidal action of complement; however, the role of Neu5Ac in this phenomenon is not clear yet. Figure 3 presents the putative roles of Neu5Ac-containing LPS in the regulation of the complement activity. Sialic acid can bind to complement factor H, which inhibits the alternative pathway of complement activation (Ram et al., 1998; Blaum et al., 2015). One of our first studies did not confirm Ram’s findings cited above. Bugla-Płoskońska et al. studied different species representing *Enterobacteriaceae* family—all containing sialic acid in LPS. Bovine blood serum was used for testing, and the results showed that all tested strains were killed *via* the alternative complement pathway activation without contribution of antibodies (Bugla-Płoskońska and Doroszkiewicz, 2006). *Enterobacteriaceae* are usually opportunistic pathogens; however, there are also primary pathogens in the family, e.g., *Salmonella*. Among the *Salmonella* genus one serogroup: O48 contains sialic acid in its O-Ag structure. Most of our studies are based on *Salmonella* O48 pathogenicity connected with the presence of Neu5Ac in LPS (Bugla-Płoskońska and Doroszkiewicz, 2006; Bugla-Płoskońska et al., 2010a,b; Pawlak et al., 2017).

In further experiments, our team established 3 mechanisms of complement activation leading to bactericidal effect on *Salmonella* O48 cells. The experiments also confirmed our previous findings that the mere Neu5Ac presence in LPS of tested strains is not sufficient for blocking the alternative pathway of complement activation (Bugla-Płoskońska et al., 2010a). The study of Bugla-Płoskońska et al. (2010b) has shown that serovars of *Salmonella* O48, regardless of the same structure of LPS O-Ag, showed high variability in the amount of sialic acid in LPS. Nevertheless, the length of O-Ag of the serovars did not directly correlate with bacterial cells’ susceptibility to human blood serum action. In subsequent studies of Pawlak et al. (2017), when we examined selected serovars of strains tested previously, the bacterial cells were repeatedly treated with human serum. The results showed that, after subsequent passages in the serum, the Neu5Ac/Kdo ratio in bacteria was considerably increased, which correlated with gradual rise of bacteria resistance. This strongly suggests that bacteria with LPS decorated with Neu5Ac can avoid the action of complement if it possesses the ability to increase the average length of LPS O-Ag on its surface.

Little is known about the influence of sialylated bacterial structures on C3 protein fixation in serum. The first analysis of C3 activation on *Salmonella* isolates of the O48 serogroup, was performed by Futoma-Kołoch et al. Investigating the relation between the amount of Neu5Ac in LPS and C3 complement protein activation in serum, authors demonstrated that the greatest C3 fragments deposition occurred on *Salmonella* Isaszeg characterized by a low content of sialic acid in LPS. In turn, C3 deposition ratio on the *Salmonella* Ngozi and *Salmonella* subsp. *arizonae,* with high contents of sialic acid in LPS, was weaker and correlated with the lower C3 activation by their LPSs. Remarkably, additional analysis performed using immunoblotting revealed that outer membrane proteins isolated from the tested strains also bound C3 protein fragments independently of LPS (Futoma-Kołoch et al., 2015).

Besides *Salmonella* O48, also the other, highly pathogenic bacterial species, utilize Neu5Ac to increase their virulence. According to some researchers (Gulati et al., 2015), the resistance of *N. gonorrhoeae* to serum is Neu5Ac-dependent but also requires the presence of PorB. This shows the multifactorial basis of serum resistance phenomenon. The most recent studies demonstrate that inhibition of sialic acid utilization by *H. influenzae* enhanced serum-mediated killing. The symbiotic strains of *H. influenzae* do not possess the ability to utilize host sialic acid (Heise et al., 2018). However, Keo et al. showed that for *C. jejuni*, capsule expression was essential for serum resistance and LOS plays role in protecting bacterial cell from cationic antimicrobial peptides and proteins (Keo et al., 2011). Nevertheless, the presence of Neu5Ac in LOS is an important virulence factor empowering the bacteria to be opportunistic pathogens. The pathways, used by pathogens to utilize the sialic acid and avoid the action of the complement, seem to be very promising therapeutic targets (Heise et al., 2018; Moons et al., 2021).

Toll-like receptors (TLRs) are proteins expressed on cells of immune system like macrophages or dendritic cells, serving as pattern-recognition receptors, an important component of the innate immune system. This class of receptors recognizes specific structures on microbial cells or viral particles, triggering the immune response and initiating pro-inflammatory signal transduction. TLR4 specifically recognizes, among other pathogen components, bacterial lipopolysaccharide, by its most conservative structure: lipid A (Vaure and Liu, 2014). LPS is extracted from the serum by the LPS binding protein (LBP), then presented *via* CD-14 to the TLR4-MD-2 receptor complex.

There are some results, concerning the recognition of sialylated lipooligosaccharide by TLR4. It has been shown, that for *C. jejuni* the sialylation of LOS enhances human DC activation and subsequent B cell proliferation. That effect may contribute to the development of autoimmune anti-ganglioside antibodies found in GBS patients after *C. jejuni* infection (Godschalk et al., 2004; Kuijf et al., 2010), also may affect the severity of gastro-enteritis and reactive arthritis (Mortensen et al., 2009). On the contrary, the sialylation of the *N. meningitidis* lipooligosaccharide do not affect pro-inflammatory response to the bacteria mediated by TLR4/MD2 (Pridmore et al., 2003), although mutants without lipid A, but with polysialic acid capsule, do not activate TLR4 pathway at all, while activating the immune response by TLR2 (Pridmore et al., 2001). These unequivocal results suggest, that the TLR4 recognition of sialylated lipooligosaccharides, where sialic acid is presented in terminal position of the molecule, can be structure dependent. There are no results up to date, concerning the TLR4 recognition of sialic acid-containing lipopolysaccharides. Lipopolysaccharides and lipooligosaccharides differ in the location of the sialic acid residues. In LPS most of the sialic acid is located inside the O-Ag, while only small portion of Sias are unsubstituted and terminal (located at the end of the O-Ag chain) sialic acid residues. In LOS most Sia residues are terminal and unsubstituted, also they are spatially much closer to the lipid A, than in LPS. As lipid A is the pathogen-associated molecular pattern (PAMP) recognized and bound by LPB, it can be expected, that the modification of TLR4-mediated reaction by sialic acid could be more distinct in LOS than in LPS. The structure of lipid A greatly affects the LPS recognition by TLR4 (Maeshima and Fernandez, 2013), this effect should be taken into account in future studies, comparing the effects of TLR4-mediated pro-inflammatory signal transduction between LPS and LOS from different bacteria.

The presented results indicate that the resistance of bacteria containing Neu5Ac in LPS to innate immunity system: bactericidal effect of blood serum and the TLR-mediated cellular response is not fully understood phenomenon. We documented that the presence of Neu5Ac in bacteria’s outer membrane is not sufficient to protect the cells from the lytic activity of serum. The phenomenon is multifactorial, demonstrating both the role of elongation of O-Ag and remodeling of the outer membrane proteins composition.

## Sources of acquisition of Neu5Ac by bacterial pathogens

**Figure 4:**
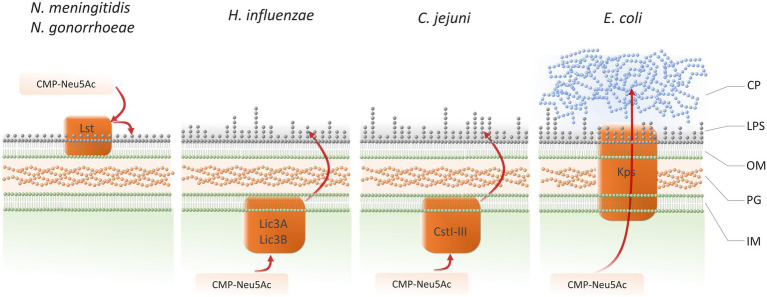
Pathways for deploying sialic acid on bacterial pathogens cell surface: *Neisseria meningitidis*, *Neisseria gonorrhoeae* (Shell et al., 2002; Lewis et al., 2015), *Haemophilus influenzae* (Jessamine and Ronald, 1990; Fox et al., 2006), *Campylobacter jejuni* (Chiu et al., 2007), *Escherichia coli* (Shen et al., 1999; Vimr et al., 2004; Severi et al., 2007). IM, inner membrane; PG, peptidoglycan; OM, outer membrane; LPS, lipopolysaccharide; CP, capsular polysaccharide.

Sialic acid is crucial for pathogenesis of some bacteria. Microbes can either synthesize sialic acid or use sialic acid of the host for metabolism as well as for the cell surface decoration (Figure 4). Free sialic acid (released by hydrolysis to the environment), can be used by bacteria in catabolic fermentation or oxidation (Vimr, 2013). Some bacteria such as *E. coli* K1, certain serotypes of *N. meningitidis* (Vimr, 2013), *Fusobacterium* (Lewis et al., 2016), or *Campylobacter jejuni* (Thomas, 2016) are able to synthesize sialic acid *de novo,* independently from the host. Other species, like *H. influenzae, N. gonorrhoeae*, *Ruminococcus gnavus,* or *Pasteurella multocida* can decorate their surface with sialic acid using sialyltransferase or hybrid synthetic-catabolic pathways (Vimr et al., 2004; Bell et al., 2020). Examples of bacteria with sialic acid-containing structures on their surface are presented in Table 1. Decoration of the surface with sialic acid was proved to be essential in pathogenesis (Vimr et al., 2000; Steenbergen et al., 2005). There is a variety of studies showing that the metabolism of sialic acid plays an important role in the process of colonization. For some species like *Streptococcus pneumoniae* (Trappetti et al., 2009) or *P. aeruginosa* (Soong, 2006) host-derived sialic acid is an important factor for biofilm formation. Some species such as *E. coli* K12 can use sialic acid as the sole source of carbon (Vimr and Troy, 1985). The utilization of the host-derived sialic acid by bacteria requires different types of transporters into the bacterial cell. The first family of transporters is major facilitator superfamily (MFS). The MFS as sialic acid transporters were firstly reported to be present at *E. coli* K12 (Vimr and Troy, 1985; Thomas, 2016). *YjhBC* operon of the *E. coli* is controlled by the repressor protein nanR, which regulates the core machinery responsible for the import and catabolic processing of sialic acid. YjhC protein is broadly involved in carbohydrate metabolism and is an oxidoreductase/dehydrogenase involved in bacterial sialic acid degradation (Horne et al., 2020).

**Table 1:** Examples of bacteria with sialic acid present on their cell surface.

Bacteria	Major disease	List of transporters	References
*Neisseria meningitidis* B	Meningitis	Kps	Vimr et al. (2004), Severi et al. (2007), and Haines-Menges et al. (2015)
*Escherichia coli* K1	Neonatal meningitis	NanT	Vimr et al. (2004) and Haines-Menges et al. (2015)
*Campylobacter jejuni*	Enteritis	Kps	Vimr et al. (2004) and Severi et al. (2007)
*Fusobacterium nucleatum*	Periodontal diseases	SiaP	Gangi Setty et al. (2014) and Thomas (2016)
*Tannerella forsythia*	Periodontal diseases	NanT	Thomas (2016) and Roy et al. (2010)
*Haemophilus influenzae*	Respiratory infections	SiaP	Gangi Setty et al. (2014), Haines-Menges et al. (2015), and Thomas (2016)
*Haemophilus ducreyi*	Chancroid	Sat-type ABC	Post et al. (2005) and Thomas (2016)
*Pseudomonas aeruginosa*	Pneumonia	NanT	Almagro-Moreno and Boyd (2010) and Green et al. (2018)
*Escherichia coli* K12	Neonatal meningitis	NanT	Vimr et al. (2004) and Haines-Menges et al. (2015)
*Salmonella* sp.	Salmonellosis	NanT, SiaT	Haines-Menges et al. (2015) and Thomas (2016)
*Vibrio cholerae*	Cholera	SiaP	Vimr et al. (2004), Gangi Setty et al. (2014), Haines-Menges et al. (2015), and Thomas (2016)
*Yersinia* sp.	Yersiniosis	NanT	Vimr et al. (2004), Haines-Menges et al. (2015), and Thomas (2016)
*Citrobacter* sp.	Infant meningitis, urinary tract infections	NanT	Thomas (2016)

One of the transporters playing important role in sialic acid transport to bacterial cell is NanT (*N*-acetylneuraminic transporter) and its homologs. These transporters are present in human pathogens like *Salmonella, Yersinia, Citrobacter*, *Chromobacter*, *Tanerella forsythia,* and also in the gut bacteria *Bacteroides fragilis*. *H. influenzae* can use host-derived sialic acid to decorate its LOS. This is necessary for innate immune evasion. However the transport mechanism is different than using NanT, and in this case, it is a tripartite ATP-independent periplasmic (TRAP) transporter (Thomas, 2016). The system with a membrane domain SiaPQM, uses SiaP, which is a substrate-binding protein (SBP). An orthologous SiaPQM system is found in pathogens like *P. multocida, Vibrio vulnificus, Vibrio cholerae, H. ducreyi, Haemophilus somnus, Actinobacillus pleuropneumoniae,* and *Corynebacterium glutamicum*, which use sat-type ABC transporter. The second group of ABC transporters involved in transport of host-derived sialic acid found in *S. pneumoniae* is satABC. The third group of transporters involved in utilization of sialic acid by *Salmonella* Typhimurium, *Vibrio fisheri*, and *Lactobacillus sakei* is sodium solute symporter family (SSS) represented by SiaT system (Thomas, 2016). Noteworthy, transporters’ families differ in the source of energy. The ABC transporters family, are primary transporters, getting energy from ATP hydrolysis. The MFS, TRAP, and SSS systems are secondary transporters using membrane potential as a source of energy, though they differ in ions required for this process (Thomas, 2016).

The usage of sialic acid for nutrition and surface decoration might be one of the factors characterizing bacterial niche specialization or disease potential. High content of Sias in human mucosal surfaces, e.g., of the gastrointestinal tract can explain, why bacteria colonize only certain areas of human body (Vimr, 2013).

After the biosynthesis or the uptake from the environment, sialic acid can be incorporated into structures on the surface of the bacterial cell, modulating the pathogen’s interaction with the host. The first step in this process is the conversion of Sia into its active form: CMP-sialic acid, which is then attached to the appropriate acceptors by means of specific sialyltransferases. Reaction is catalyzed by CMP systolic acid synthesis (Severi et al., 2007). Most of the information about the incorporation of sialic acid into outer structures of bacteria comes from the analysis of the capsule biosynthesis processes in *E. coli* and *N. meningitidis*. In *E. coli*, the NeuA protein activates Neu5Ac before being incorporated into capsule, whereas in the case of *N. meningitidis* NeuA activates Neu5Ac for capsule and LPS biosynthesis (Vimr et al., 2004).

*Neisseria meningitidis* and *N. gonorrhoeae* perform the sialylation of LOS with the participation of α-2,3-sialyltransferase (Lst). Lst protein is located in the outer membrane (Shell et al., 2002; Lewis et al., 2015). Such sialylation is also a feature of several pathogenic bacterial strains, e.g., *H. influenzae, C. jejuni* and *Helicobacter* sp. Sialyltransferases identified in these pathogens belong to glycosyltransferase family GT-42 (Audry et al., 2011). The main sialyltransferase of *H. influenzae* is Lic3A, responsible for the addition of Neu5Ac to a lactose acceptor. Lic3B α,2,3/α,2,8-sialyltransferase is a bifunctional enzyme, its activity enables the utilization of both: terminal galactose of lactose as well as Neu5Ac of sialyllactose as acceptor molecules (Jessamine and Ronald, 1990; Fox et al., 2006). The terminal sialic acid residue in the double sialylated LOS may also be modified by *O-*acetyltransferases. Sialic acid in the *O-*acetylated form can also be included as part of the O-specific chain oligosaccharides in *E. coli*, but the enzymes required for this process are not well characterized (van der Woude and Baumler, 2004; Ali et al., 2006; Severi et al., 2007). The first functionally characterized sialyltransferase of the *Helicobacter* genus is monofunctional LPS α,2,3-sialyltransferase of *H. bizzozeronii*, which has a strong preference for LacNAc (Kondadi et al., 2012). *Campylobacter jejuni* has three functional sialyltransferases from group Cst: CstI, CstII, and CstIII. CstI is the monofunctional enzyme that has been shown to utilize solely Gal-β-1,3/4-R as the acceptor sugar in its transferase reaction. It has been found, that CstII can be the bifunctional enzyme, using both Gal-β-1,3/4-R and Neu5Ac-α-2,3-Gal-β-1,3/4-R as acceptor sugars (Gilbert et al., 2002; Chiu et al., 2007).

In *E. coli*, the main α-2,8/α-2,9-polysialyltransferase, which adds Neu5Ac to the appropriate receptors to form the capsule, is the NeuS protein, which is then exported outside the cell using the Kps system (Shen et al., 1999; Vimr et al., 2004; Severi et al., 2007). Synthesized sialic acid, which is a component of the capsular polysaccharide or LPS, can be modified by *O-*acetylation. In *E. coli*, NeuO and NeuD *O-*acetyltransferases modify the capsular sialic acid, which can also be deacetylated by the NeuA protein. These studies highlight the huge functional diversity in the structure of bacterial capsules that are presented on the cell surface by numerous patterns of acetylated sialic acid (Claus et al., 2004; Steenbergen et al., 2006).

Some bacteria, such as *H. influenzae* or *E. coli*, consume transported sialic acid as a source of carbon and nitrogen (Severi et al., 2005; Kentache et al., 2021). The sialyl catabolism pathway is well characterized and is based on the cleavage of Neu5Ac to ManNAc and pyruvate by means of NanA aldolase. ManNAc is finally transformed into Fru-6P and ammonia with the participation of NanK, NanE, NagB, and NagA proteins. It is not fully elucidated how bacteria maintain the balance between catabolism and anabolism of sialic acid and how catabolism of Neu5Ac effects on the bacterial virulence (Shen et al., 1999; Vimr et al., 2004).

## Sialic acid liberation from the host by bacterial sialidases

The balance between gut microbes and secreted mucus affects intestinal health (Robinson et al., 2017). As indicated in the previous sections, the role of sialic acids is crucial in the biology of higher organisms and pathobiology of some bacteria, parasites and even viruses. Sialic acids, decorating the outermost regions of carbohydrates in host glycoproteins, may be utilized by microorganisms. It is well documented that enteric bacteria, both commensal and pathogenic, can use host sialic acids as a nutrient source, thus unmasking host ligands used for adherence or contribute in biofilm formation (Juge et al., 2016). For these purposes, bacteria express secretory proteins called sialidases or neuraminidases that hydrolyze host sialic acid residues (Lewis and Lewis, 2012).

Remarkably, it was found that *O-*acetyl ester modifications of sialic acids improve the resistance against the action of a number of sialidases, some gut bacteria (e.g., *Bacteroides*), in turn, produce sialate-*O-*acetylesterases to remove the ester group (Robinson et al., 2017), which improves the accessibility of sialic acids to gut bacterial sialidases. The group of sialidases (EC 3.2.1.18) belongs to a class of glycosyl hydrolases which remove terminal *N*-acylneuraminate residues from the glycans of glycoproteins, glycolipids, and polysaccharides. Bacterial sialidases can catalyze the hydrolysis of terminal sialic acids linked by the α-2,3-, α-2,6- or α-2,8-linkage to a diverse assortment of substrates. Besides, some of these enzymes can catalyze the transfer of sialic acids from sialoglycans to asialoglycoconjugates *via* a transglycosylation reaction mechanism (Robinson et al., 2017).

The most frequently defined enteric bacteria, producing sialidases, are *Bacteroides*, *Prevotella, Clostridium*, *Bifidobacterium longum*, *V. cholerae* (Fraser, 1978; Moncla et al., 1990; Moustafa et al., 2004; Sela et al., 2008), and *S.* Typhimurium (Corfield et al., 1983; Kim et al., 2011). The production of sialidases by *Salmonella* sp. is not widespread, and only one sialidase from *S.* Typhimurium has been functionally characterized (Juge et al., 2016). Other studies show that *S.* Typhimurium encodes the *nan* operon, but lacks the sialidase required for sialic acid release (Miller and Hoskins, 1981; Corfield et al., 1992). Among *E. coli* strains, enteropathogenic *E. coli* O127 strain (EPEC; Iguchi et al., 2009) possess a sialidase-encoding gene, whereas commensal *E. coli* strains such as *E. coli* strain EHV2 lack a sialidase (Huang et al., 2015; Juge et al., 2016), what suggests, that the production of sialidases is connected with the pathogenicity of microorganism.

Recently published research describes that problem in organisms inhabiting oral cavity, for instance *T. forsythia* (Frey et al., 2018) or *Porphyromonas gingivalis* (Xu et al., 2017; Yang et al., 2018). *T. forsythia* is a key organism in periodontal disease with the ability of sialic acid utilization (Frey et al., 2018). Yang et al. found that *P. gingivalis* sialidase gene mutant strain showed less pathogenicity than the wild-type strain. Indeed, research on the *P. gingivalis* interaction with the host phagocytes showed that inhibition of sialidase in bacteria led to rapid clearance of pathogens by macrophages (Yang et al., 2018). Other studies with the same species revealed that the cell membrane of the sialidase-deficient mutant was more sensitive to a damage caused by oxidative stress. Moreover, sialylation of the components on the *P. gingivalis* cell surface helped in biofilm formation. The authors implied that the inhibition of *P. gingivalis* sialidase, using a sialidase inhibitor, would reduce pathogen survival, virulence and biofilm formation, what can facilitate the therapy of periodontal disease (Xu et al., 2017). Activity of *P. gingivalis* sialidase can be inhibited using the commercially available drug zanamivir, what leads to an inhibition of *P. gingivalis* biofilm formation on oral glycoprotein sources, also inhibited attachment and invasion by *P. gingivalis*, *T. forsythia,* and other periodontal pathogens to oral epithelial cells (Honma et al., 2011; Frey et al., 2019). Other anaerobic oral bacteria associated with periodontitis, *F. nucleatum*, also have a sialic acid as a component of the O-antigen LPS. *T. forsythia*, which is found in close association with *F. nucleatum*, could harvest sialic acid from *F. nucleatum* LPS. Sialic acid can be catabolized as a source of carbon and growth factor by cohabiting species of the dental plaque and likely plays a role in the dental biofilm development (Vinogradov et al., 2017).

Lewis and Lewis focused on the way how sialidases action affects pathogenic, commensal and/or symbiotic host–microbe interactions (Lewis and Lewis, 2012). In addition Wong et al. (2018) show the intriguing connection between two species of bacteria: *Streptococcus oralis* and *Streptococcus gordonii.* It was documented that *S. oralis* increases *S. gordonii* adherence in a neuraminidase-dependent manner. It is important to emphasize that sialidases produced by the microbiota in gut may promote the expansion of some potential antibiotic-associated pathogens, including *E. coli*, *Clostridium difficile,* and *Salmonella* sp. pathotypes that do not produce these enzymes. The cross-species feeding on the host sialic acids between members of the gut or oral cavity microbiomes may have a significant contribution in the infection process (Robinson et al., 2017).

## Conclusion

The role of sialic acids in higher organisms is very wide. As very versatile modulators of cell functions and pathology, sialic acids participate in diverse cellular mechanisms. They are used mostly for recognition purposes: being a ligand for a great variety of molecules as lectins, antibodies or hormones, and also as an agent, masking biological recognition sites. The important role of host sialic acids in immune reactions, cell differentiation or apoptosis is still the subject of analysis. With such a wide array of functions for that class of molecules it is not surprising, that the bacteria can utilize sialic acids, like Neu5Ac, for the modification of its outer structures, which then interact with the natural functions of the host in multiple ways. Several examples of this interaction, like molecular mimicry, liberation, and utilization of host sialic acid by bacteria, the influence on bacterial pathogenicity, or the etiology of autoimmunological diseases are presented in this work. Moreover, the structural relationship between sialic acids and the sugars, which are found mostly in bacterial endotoxins: 2-keto-3-deoxy-nonulosonic acid (Kdn) and 2-keto-3-deoxy-oculosonic acid (Kdo), can raise questions about the possibilities of new and yet unexplored interactions between bacterial endotoxins and host sialic acid recognition systems, which definitely will be the subject of further intensive studies in future.

## Author contributions

BD, JR, GB-P, AK-K, BF-K, AP, and AG wrote, edited, and reviewed the manuscript. BD, JR, and AG prepared the figures and tables. All authors contributed to the article and approved the submitted version.

## Funding

This work partially gained knowledge from research project “The strategy of *Salmonella* O48 having sialylated lipopolysaccharides to avoid complement-driven immune response” (BF-K) supported by program “Excellence Initiative-Research University (IDUB)” for years 2020–2026 for the University of Wrocław, Poland.

## Conflict of interest

The authors declare that the research was conducted in the absence of any commercial or financial relationships that could be construed as a potential conflict of interest.

## Publisher’s note

All claims expressed in this article are solely those of the authors and do not necessarily represent those of their affiliated organizations, or those of the publisher, the editors and the reviewers. Any product that may be evaluated in this article, or claim that may be made by its manufacturer, is not guaranteed or endorsed by the publisher.

## References

[ref1] Albert L. J. Inman R. D. (1999). Molecular mimicry and autoimmunity. N. Engl. J. Med. 341, 2068–2074. doi: 10.1056/NEJM199912303412707 10615080

[ref2] Ali T. Weintraub A. Widmalm G. (2006). Structural determination of the O-antigenic polysaccharide from the Shiga toxin-producing *Escherichia coli* O171. Carbohydr. Res. 341, 1878–1883. doi: 10.1016/j.carres.2006.04.002, PMID: 16697991

[ref3] Almagro-Moreno S. Boyd E. F. (2010). Bacterial catabolism of nonulosonic (sialic) acid and fitness in the gut. Gut Microbes 1, 45–50. doi: 10.4161/gmic.1.1.10386, PMID: 21327116 PMC3035139

[ref4] Aspinall G. O. Fujimoto S. McDonald A. G. Pang H. Kurjanczyk L. A. Penner J. L. (1994). Lipopolysaccharides from *campylobacter jejuni* associated with Guillain-Barré syndrome patients mimic human gangliosides in structure. Infect. Immun. 62, 2122–2125. doi: 10.1128/iai.62.5.2122-2125.1994, PMID: 8168981 PMC186479

[ref5] Audry M. Jeanneau C. Imberty A. Harduin-Lepers A. Delannoy P. Breton C. (2011). Current trends in the structure-activity relationships of sialyltransferases. Glycobiology 21, 716–726. doi: 10.1093/glycob/cwq189, PMID: 21098518

[ref6] Avril T. Wagner E. R. Willison H. J. Crocker P. R. (2006). Sialic acid-binding immunoglobulin-like lectin 7 mediates selective recognition of sialylated glycans expressed on *campylobacter jejuni* lipooligosaccharides. Infect. Immun. 74, 4133–4141. doi: 10.1128/IAI.02094-05, PMID: 16790787 PMC1489752

[ref7] Barry G. T. (1958). Colominic acid, a polymer of N-acetylneuraminic acid. J. Exp. Med. 107, 507–521. doi: 10.1084/jem.107.4.507, PMID: 13513915 PMC2136837

[ref8] Barry G. T. (1959). Detection of sialic acid in various *Escherichia coli* strains and in other species of bacteria. Nature 183, 117–118. doi: 10.1038/183117a0, PMID: 13622714

[ref9] Bell A. Severi E. Lee M. Monaco S. Latousakis D. Angulo J.. (2020). Uncovering a novel molecular mechanism for scavenging sialic acids in bacteria. J. Biol. Chem. 295, 13724–13736. doi: 10.1074/jbc.RA120.014454, PMID: 32669363 PMC7535918

[ref10] Bhattacharjee A. K. Jennings H. J. Kenny C. P. Martin A. Smith I. C. (1975). Structural determination of the sialic acid polysaccharide antigens of *Neisseria meningitidis* serogroups B and C with carbon 13 nuclear magnetic resonance. J. Biol. Chem. 250, 1926–1932. doi: 10.1016/S0021-9258(19)41784-5, PMID: 163259

[ref11] Blaum B. S. Hannan J. P. Herbert A. P. Kavanagh D. Uhrín D. Stehle T. (2015). Structural basis for sialic acid–mediated self-recognition by complement factor H. Nat. Chem. Biol. 11, 77–82. doi: 10.1038/nchembio.1696, PMID: 25402769

[ref12] Bolaños R. DeWitt C. W. (1966). Isolation and characterization of the K1 (L) antigen of *Escherichia coli*. J. Bacteriol. 91, 987–996. doi: 10.1128/jb.91.3.987-996.1966, PMID: 5326105 PMC315989

[ref13] Boleij M. Kleikamp H. Pabst M. Neu T. R. van Loosdrecht M. C. M. Lin Y. (2020). Decorating the anammox house: sialic acids and sulfated glycosaminoglycans in the extracellular polymeric substances of anammox granular sludge. Environ. Sci. Technol. 54, 5218–5226. doi: 10.1021/acs.est.9b07207, PMID: 32227885 PMC7181257

[ref14] Bravo D. Silva C. Carter J. A. Hoare A. Alvarez S. A. Blondel C. J.. (2008). Growth-phase regulation of lipopolysaccharide O-antigen chain length influences serum resistance in serovars of *salmonella*. J. Med. Microbiol. 57, 938–946. doi: 10.1099/jmm.0.47848-0, PMID: 18628492

[ref15] Bugla-Płoskońska G. Doroszkiewicz W. (2006). Bactericidal activity of normal bovine serum (NBS) directed against some *Enterobacteriaceae* with sialic acid-containing lipopolysaccharides (LPS) as a component of cell wall. Pol. J. Microbiol. 55, 169–174. 17338268

[ref16] Bugla-Płoskońska G. Futoma-Kołoch B. Rybka J. Gamian A. Doroszkiewicz W. (2010a). The role of complement activity in the sensitivity of *salmonella* O48 strains with sialic acid-containing lipopolysaccharides to the bactericidal action of normal bovine serum. Pol. J. Vet. Sci. 13, 53–62. 21077431

[ref17] Bugla-Płoskońska G. Rybka J. Futoma-Kołoch B. Cisowska A. Gamian A. Doroszkiewicz W. (2010b). Sialic acid-containing lipopolysaccharides of *salmonella* O48 strains—potential role in camouflage and susceptibility to the bactericidal effect of normal human serum. Microb. Ecol. 59, 601–613. doi: 10.1007/s00248-009-9600-2, PMID: 19844648

[ref18] Carlin A. F. Lewis A. L. Varki A. Nizet V. (2007). Group B streptococcal capsular sialic acids interact with siglecs (immunoglobulin-like lectins) on human leukocytes. J. Bacteriol. 189, 1231–1237. doi: 10.1128/JB.01155-06, PMID: 16997964 PMC1797352

[ref19] Chang Y.-C. Nizet V. (2014). The interplay between Siglecs and sialylated pathogens. Glycobiology 24, 818–825. doi: 10.1093/glycob/cwu067, PMID: 24996821 PMC4168292

[ref20] Chiu C. P. C. Lairson L. L. Gilbert M. Wakarchuk W. W. Withers S. G. Strynadka N. C. J. (2007). Structural analysis of the alpha-2,3-sialyltransferase Cst-I from *campylobacter jejuni* in apo and substrate-analogue bound forms. Biochemistry 46, 7196–7204. doi: 10.1021/bi602543d, PMID: 17518445

[ref21] Claus H. Borrow R. Achtman M. Morelli G. Kantelberg C. Longworth E.. (2004). Genetics of capsule O-acetylation in serogroup C, W-135 and Y meningococci. Mol. Microbiol. 51, 227–239. doi: 10.1046/j.1365-2958.2003.03819.x, PMID: 14651624

[ref22] Corfield A. P. Higa H. Paulson J. C. Schauer R. (1983). The specificity of viral and bacterial sialidases for alpha(2-3)- and alpha(2-6)-linked sialic acids in glycoproteins. Biochim. Biophys. Acta 744, 121–126. doi: 10.1016/0167-4838(83)90080-8, PMID: 6301560

[ref23] Corfield A. P. Wagner S. A. Clamp J. R. Kriaris M. S. Hoskins L. C. (1992). Mucin degradation in the human colon: production of sialidase, sialate O-acetylesterase, N-acetylneuraminate lyase, arylesterase, and glycosulfatase activities by strains of fecal bacteria. Infect. Immun. 60, 3971–3978. doi: 10.1128/iai.60.10.3971-3978.1992, PMID: 1398908 PMC257425

[ref24] de Graaff D. R. Felz S. Neu T. R. Pronk M. van Loosdrecht M. C. M. Lin Y. (2019). Sialic acids in the extracellular polymeric substances of seawater-adapted aerobic granular sludge. Water Res. 155, 343–351. doi: 10.1016/j.watres.2019.02.040, PMID: 30852321

[ref25] Dewitt C. W. Rowe J. A. (1961). Sialic acids (N,7-O-diacetylneuraminic acid and N-acetylneuraminic acid) in *Escherichia coli* I. Isolation and identification. J. Bacteriol. 82, 838–848. doi: 10.1128/jb.82.6.838-848.1961, PMID: 13885921 PMC279266

[ref26] Dudek B. Krzyżewska E. Kapczyńska K. Rybka J. Pawlak A. Korzekwa K.. (2016). Proteomic analysis of outer membrane proteins from *salmonella* Enteritidis strains with different sensitivity to human serum. PLoS One 11:e0164069. doi: 10.1371/journal.pone.0164069, PMID: 27695090 PMC5047454

[ref27] Ebaid H. Duk M. Gamian A. (2008). Antibodies against *Citrobacter braakii* O37 cells recognize the *N* -glycan of the band 3 glycoprotein of human erythrocyte membrane. FEMS Immunol. Med. Microbiol. 52, 352–361. doi: 10.1111/j.1574-695X.2008.00380.x, PMID: 18266742

[ref28] Feng Y. Cao M. Shi J. Zhang H. Hu D. Zhu J.. (2012). Attenuation of *Streptococcus suis* virulence by the alteration of bacterial surface architecture. Sci. Rep. 2. doi: 10.1038/srep00710, PMID: 23050094 PMC3464449

[ref29] Feng L. Senchenkova S. N. Yang J. Shashkov A. S. Tao J. Guo H.. (2004). Synthesis of the heteropolysaccharide O antigen of *Escherichia coli* O52 requires an ABC transporter: structural and genetic evidence. J. Bacteriol. 186, 4510–4519. doi: 10.1128/JB.186.14.4510-4519.2004, PMID: 15231783 PMC438562

[ref30] Figueira M. A. Ram S. Goldstein R. Hood D. W. Moxon E. R. Pelton S. I. (2007). Role of complement in defense of the middle ear revealed by restoring the virulence of nontypeable *Haemophilus influenzae* siaB mutants. Infect. Immun. 75, 325–333. doi: 10.1128/IAI.01054-06, PMID: 17088344 PMC1828410

[ref31] Fox K. L. Cox A. D. Gilbert M. Wakarchuk W. W. Li J. Makepeace K.. (2006). Identification of a bifunctional lipopolysaccharide sialyltransferase in *Haemophilus influenzae*: incorporation of disialic acid. J. Biol. Chem. 281, 40024–40032. doi: 10.1074/jbc.M602314200, PMID: 17071616

[ref32] Fraser A. G. (1978). Neuraminidase production by clostridia. J. Med. Microbiol. 11, 269–280. doi: 10.1099/00222615-11-3-269 210277

[ref33] Frey A. M. Satur M. J. Phansopa C. Honma K. Urbanowicz P. A. Spencer D. I. R.. (2019). Characterization of *Porphyromonas gingivalis* sialidase and disruption of its role in host-pathogen interactions. Microbiology 165, 1181–1197. doi: 10.1099/mic.0.000851, PMID: 31517596 PMC7137779

[ref34] Frey A. M. Satur M. J. Phansopa C. Parker J. L. Bradshaw D. Pratten J.. (2018). Evidence for a carbohydrate-binding module (CBM) of *Tannerella forsythia* NanH sialidase, key to interactions at the host–pathogen interface. Biochem. J. 475, 1159–1176. doi: 10.1042/BCJ20170592, PMID: 29483296

[ref35] Futoma-Kołoch B. Godlewska U. Guz-Regner K. Dorotkiewicz-Jach A. Klausa E. Rybka J.. (2015). Presumable role of outer membrane proteins of *salmonella* containing sialylated lipopolysaccharides serovar Ngozi, sv. Isaszeg and subspecies arizonae in determining susceptibility to human serum. Gut Pathog. 7:18. doi: 10.1186/s13099-015-0066-0, PMID: 26185527 PMC4504086

[ref36] Gamian A. Beurret M. Michon F. Brisson J. R. Jennings H. J. (1992a). Structure of the L2 lipopolysaccharide core oligosaccharides of *Neisseria meningitidis*. J. Biol. Chem. 267, 922–925. doi: 10.1016/S0021-9258(18)48372-X, PMID: 1730681

[ref37] Gamian A. Jones C. Lipiński T. Korzeniowska-Kowal A. Ravenscroft N. (2000). Structure of the sialic acid-containing O-specific polysaccharide from *salmonella* enterica serovar Toucra O48 lipopolysaccharide: sialic-acid-containing polysaccharide of S. Toucra O48. Eur. J. Biochem. 267, 3160–3167. doi: 10.1046/j.1432-1327.2000.01335.x, PMID: 10824100

[ref38] Gamian A. Kenne L. (1993). Analysis of 7-substituted sialic acid in some enterobacterial lipopolysaccharides. J. Bacteriol. 175, 1508–1513. doi: 10.1128/jb.175.5.1508-1513.1993, PMID: 8444811 PMC193239

[ref39] Gamian A. Kenne L. Mieszała M. Ulrich J. Defaye J. (1994). Structure of the *Escherichia coli* O24 and O56 O-specific sialic-acid-containing polysaccharides and linkage of these structures to the core region in lipopolysaccharides. Eur. J. Biochem. 225, 1211–1220. doi: 10.1111/j.1432-1033.1994.1211b.x, PMID: 7525286

[ref40] Gamian A. Romanowska E. Dabrowski U. Dabrowski J. (1991). Structure of the O-specific, sialic acid containing polysaccharide chain and its linkage to the core region in lipopolysaccharide from *hafnia alvei* strain 2 as elucidated by chemical methods, gas-liquid chromatography/mass spectrometry, and 1H NMR spectroscopy. Biochemistry 30, 5032–5038. doi: 10.1021/bi00234a027 2036370

[ref41] Gamian A. Romanowska A. Romanowska E. (1992b). Immunochemical studies on sialic acid-containing lipopolysaccharides from enterobacterial species. FEMS Microbiol. Immunol. 4, 323–328. doi: 10.1111/j.1574-6968.1992.tb05012.x, PMID: 1524838

[ref42] Gamian A. Romanowska E. Ulrich J. Defaye J. (1992c). The structure of the sialic acid-containing *Escherichia col*i O104 O-specific polysaccharide and its linkage to the core region in lipopolysaccharide. Carbohydr. Res. 236, 195–208. doi: 10.1016/0008-6215(92)85016-S, PMID: 1291048

[ref43] Gangi Setty T. Cho C. Govindappa S. Apicella M. A. Ramaswamy S. (2014). Bacterial periplasmic sialic acid-binding proteins exhibit a conserved binding site. Acta Crystallogr. D Biol. Crystallogr. 70, 1801–1811. doi: 10.1107/S139900471400830X, PMID: 25004958 PMC4089482

[ref44] Gilbert M. Karwaski M. F. Bernatchez S. Young N. M. Taboada E. Michniewicz J.. (2002). The genetic bases for the variation in the lipo-oligosaccharide of the mucosal pathogen, *campylobacter jejuni*. Biosynthesis of sialylated ganglioside mimics in the core oligosaccharide. J. Biol. Chem. 277, 327–337. doi: 10.1074/jbc.M108452200, PMID: 11689567

[ref45] Godschalk P. C. Heikema A. P. Gilbert M. Komagamine T. Ang C. W. Glerum J.. (2004). The crucial role of *campylobacter jejuni* genes in anti-ganglioside antibody induction in Guillain-Barre syndrome. J. Clin. Invest. 114, 1659–1665. doi: 10.1172/JCI15707, PMID: 15578098 PMC529276

[ref46] Gowthaman U. Eswarakumar V. P. (2013). Molecular mimicry: good artists copy, great artists steal. Virulence 4, 433–434. doi: 10.4161/viru.25780, PMID: 23863600 PMC5359722

[ref47] Green A. E. Amézquita A. Le Marc Y. Bull M. J. Connor T. R. Mahenthiralingam E. (2018). The consistent differential expression of genetic pathways following exposure of an industrial *Pseudomonas aeruginosa* strain to preservatives and a laundry detergent formulation. FEMS Microbiol. Lett. 365. doi: 10.1093/femsle/fny062, PMID: 29548026 PMC5905593

[ref48] Gulati S. Schoenhofen I. C. Whitfield D. M. Cox A. D. Li J. S. Michael F.. (2015). Utilizing CMP-sialic acid analogs to unravel *Neisseria gonorrhoeae* Lipooligosaccharide-mediated complement resistance and design novel therapeutics. PLoS Pathog. 11:e1005290. doi: 10.1371/journal.ppat.1005290, PMID: 26630657 PMC4668040

[ref49] Gunn J. S. (2000). Mechanisms of bacterial resistance and response to bile. Microbes Infect. 2, 907–913. doi: 10.1016/S1286-4579(00)00392-0 10962274

[ref50] Hahn A. F. (1998). Guillain-Barré syndrome. Lancet Lond. Engl. 352, 635–641. doi: 10.1016/S0140-6736(97)12308-X 9746040

[ref51] Haines-Menges B. L. Whitaker W. B. Lubin J. B. Boyd E. F. (2015). Host sialic acids: a delicacy for the pathogen with discerning taste. Microbiol. Spectr. 3. doi: 10.1128/microbiolspec.MBP-0005-2014, PMID: 26350327 PMC6089508

[ref52] Hakomori S. (1981). Blood group ABH and ii antigens of human erythrocytes: chemistry, polymorphism, and their developmental change. Semin. Hematol. 18, 39–62. 6782678

[ref53] Han Z. Thuy-Boun P. S. Pfeiffer W. Vartabedian V. F. Torkamani A. Teijaro J. R.. (2021). Identification of an N-acetylneuraminic acid-presenting bacteria isolated from a human microbiome. Sci. Rep. 11:4763. doi: 10.1038/s41598-021-83875-w, PMID: 33637779 PMC7910532

[ref54] Hao Q. Saida T. Kuroki S. Nishimura M. Nukina M. Obayashi H.. (1998). Antibodies to gangliosides and galactocerebroside in patients with Guillain-Barré syndrome with preceding *campylobacter jejuni* and other identified infections. J. Neuroimmunol. 81, 116–126. doi: 10.1016/S0165-5728(97)00166-5, PMID: 9521613

[ref55] Heikema A. P. Bergman M. P. Richards H. Crocker P. R. Gilbert M. Samsom J. N.. (2010). Characterization of the specific interaction between Sialoadhesin and Sialylated *campylobacter jejuni* lipooligosaccharides. Infect. Immun. 78, 3237–3246. doi: 10.1128/IAI.01273-09, PMID: 20421384 PMC2897406

[ref56] Heise T. Langereis J. D. Rossing E. de Jonge M. I. Adema G. J. Büll C.. (2018). Selective inhibition of sialic acid-based molecular mimicry in *Haemophilus influenzae* abrogates serum resistance. Cell Chem. Biol. 25, 1279.e8–1285.e8. doi: 10.1016/j.chembiol.2018.05.018, PMID: 29983272

[ref57] Honma K. Mishima E. Sharma A. (2011). Role of *Tannerella forsythia* NanH sialidase in epithelial cell attachment. Infect. Immun. 79, 393–401. doi: 10.1128/IAI.00629-10, PMID: 21078857 PMC3019913

[ref58] Horne C. R. Kind L. Davies J. S. Dobson R. C. J. (2020). On the structure and function of *Escherichia coli* YjhC: an oxidoreductase involved in bacterial sialic acid metabolism. Proteins Struct. Funct. Bioinforma. 88, 654–668. doi: 10.1002/prot.25846 31697432

[ref59] Huang Y.-L. Chassard C. Hausmann M. von Itzstein M. Hennet T. (2015). Sialic acid catabolism drives intestinal inflammation and microbial dysbiosis in mice. Nat. Commun. 6:8141. doi: 10.1038/ncomms9141, PMID: 26303108 PMC4560832

[ref60] Huizinga R. van Rijs W. Bajramovic J. J. Kuijf M. L. Laman J. D. Samsom J. N.. (2013). Sialylation of *campylobacter jejuni* endotoxin promotes dendritic cell-mediated B cell responses through CD14-dependent production of IFN- and TNF. J. Immunol. 191, 5636–5645. doi: 10.4049/jimmunol.1301536, PMID: 24166974

[ref61] Iguchi A. Thomson N. R. Ogura Y. Saunders D. Ooka T. Henderson I. R.. (2009). Complete genome sequence and comparative genome analysis of enteropathogenic *Escherichia coli* O127:H6 strain E2348/69. J. Bacteriol. 191, 347–354. doi: 10.1128/JB.01238-08, PMID: 18952797 PMC2612414

[ref62] Jacobs B. C. Hazenberg M. P. van Doorn P. A. Endtz H. P. van der Meché F. G. (1997). Cross-reactive antibodies against gangliosides and *campylobacter jejuni* lipopolysaccharides in patients with Guillain-Barré or Miller fisher syndrome. J Infect Dis 175, 729–733. doi: 10.1093/infdis/175.3.729 9041356

[ref63] Jarvis G. A. Vedros N. A. (1987). Sialic acid of group B *Neisseria meningitidis* regulates alternative complement pathway activation. Infect. Immun. 55, 174–180. doi: 10.1128/IAI.55.1.174-180.1987, PMID: 3098684 PMC260297

[ref64] Jennings H. J. Johnson K. G. Kenne L. (1983). The structure of an R-type oligosaccharide core obtained from some lipopolysaccharides of *Neisseria meningitidis*. Carbohydr. Res. 121, 233–241. doi: 10.1016/0008-6215(83)84020-8, PMID: 6421484

[ref65] Jessamine P. G. Ronald A. R. (1990). Chancroid and the role of genital ulcer disease in the spread of human retroviruses. Med. Clin. North Am. 74, 1417–1431. doi: 10.1016/S0025-7125(16)30488-6, PMID: 2246947

[ref66] John C. M. Schneider H. Griffiss J. M. (1999). *Neisseria gonorrhoeae* that infect men have Lipooligosaccharides with terminal *N*-Acetyllactosamine repeats. J. Biol. Chem. 274, 1017–1025. doi: 10.1074/jbc.274.2.1017, PMID: 9873046

[ref67] Jones C. Virji M. Crocker P. R. (2003). Recognition of sialylated meningococcal lipopolysaccharide by siglecs expressed on myeloid cells leads to enhanced bacterial uptake. Mol. Microbiol. 49, 1213–1225. doi: 10.1046/j.1365-2958.2003.03634.x, PMID: 12940982

[ref68] Juge N. Tailford L. Owen C. D. (2016). Sialidases from gut bacteria: a mini-review. Biochem. Soc. Trans. 44, 166–175. doi: 10.1042/BST20150226, PMID: 26862202 PMC4747158

[ref69] Kentache T. Thabault L. Deumer G. Haufroid V. Frédérick R. Linster C. L.. (2021). The metalloprotein YhcH is an anomerase providing N-acetylneuraminate aldolase with the open form of its substrate. J. Biol. Chem. 296:100699. doi: 10.1016/j.jbc.2021.100699, PMID: 33895133 PMC8141875

[ref70] Keo T. Collins J. Kunwar P. Blaser M. J. Iovine N. M. (2011). *Campylobacter* capsule and lipooligosaccharide confer resistance to serum and cationic antimicrobials. Virulence 2, 30–40. doi: 10.4161/viru.2.1.14752, PMID: 21266840 PMC3073237

[ref71] Khatua B. Ghoshal A. Bhattacharya K. Mandal C. Saha B. Crocker P. R.. (2010). Sialic acids acquired by *Pseudomonas aeruginosa* are involved in reduced complement deposition and siglec mediated host-cell recognition. FEBS Lett. 584, 555–561. doi: 10.1016/j.febslet.2009.11.087, PMID: 19945458 PMC3640159

[ref72] Kim S. Oh D.-B. Kang H. A. Kwon O. (2011). Features and applications of bacterial sialidases. Appl. Microbiol. Biotechnol. 91, 1–15. doi: 10.1007/s00253-011-3307-2, PMID: 21544654

[ref73] Kondadi P. K. Rossi M. Twelkmeyer B. Schur M. J. Li J. Schott T.. (2012). Identification and characterization of a lipopolysaccharide α,2,3-sialyltransferase from the human pathogen *helicobacter bizzozeronii*. J. Bacteriol. 194, 2540–2550. doi: 10.1128/JB.00126-12, PMID: 22408169 PMC3347187

[ref74] Korzeniowska-Kowal A. Kochman A. Gamian E. Lis-Nawara A. Lipiński T. Seweryn E.. (2015). Antibodies against *Escherichia coli* O24 and O56 O-specific polysaccharides recognize epitopes in human glandular epithelium and nervous tissue. PLoS One 10:e0129492. doi: 10.1371/journal.pone.0129492, PMID: 26086646 PMC4472344

[ref75] Korzeniowska-Kowal A. Witkowska D. Gamian A. (2001). Molecular mimicry of bacterial polysaccharides and their role in etiology of infectious and autoimmune diseases. Postepy Hig. Med. Dosw. 55, 211–232. 11468971

[ref76] Krauss J. H. Himmelspach K. Reuter G. Schauer R. Mayer H. (1992). Structural analysis of a novel sialic-acid-containing trisaccharide from *Rhodobacter capsulatus* 37b4 lipopolysaccharide. Eur. J. Biochem. 204, 217–223. doi: 10.1111/j.1432-1033.1992.tb16627.x, PMID: 1310942

[ref77] Kuijf M. L. Samsom J. N. van Rijs W. Bax M. Huizinga R. Heikema A. P.. (2010). TLR4-mediated sensing of *campylobacter jejuni* by dendritic cells is determined by sialylation. J. Immunol. 185, 748–755. doi: 10.4049/jimmunol.0903014, PMID: 20525894

[ref78] Lewis L. A. Gulati S. Burrowes E. Zheng B. Ram S. Rice P. A. (2015). α-2,3-sialyltransferase expression level impacts the kinetics of lipooligosaccharide sialylation, complement resistance, and the ability of Neisseria gonorrhoeae to colonize the murine genital tract. MBio 6. doi: 10.1128/mBio.02465-14, PMID: 25650401 PMC4324315

[ref79] Lewis A. L. Lewis W. G. (2012). Host sialoglycans and bacterial sialidases: a mucosal perspective: mucosal sialoglycans and bacterial sialidases. Cell. Microbiol. 14, 1174–1182. doi: 10.1111/j.1462-5822.2012.01807.x, PMID: 22519819

[ref80] Lewis A. L. Robinson L. S. Agarwal K. Lewis W. G. (2016). Discovery and characterization of *de novo* sialic acid biosynthesis in the phylum *fusobacterium*. Glycobiology 26, 1107–1119. doi: 10.1093/glycob/cww068, PMID: 27613803 PMC5072148

[ref81] Madico G. Ngampasutadol J. Gulati S. Vogel U. Rice P. A. Ram S. (2007). Factor H binding and function in sialylated pathogenic neisseriae is influenced by gonococcal, but not meningococcal, porin. J. Immunol. 178, 4489–4497. doi: 10.4049/jimmunol.178.7.4489, PMID: 17372007

[ref82] Maeshima N. Fernandez R. C. (2013). Recognition of lipid a variants by the TLR4-MD-2 receptor complex. Front. Cell. Infect. Microbiol. 3:3. doi: 10.3389/fcimb.2013.00003, PMID: 23408095 PMC3569842

[ref83] Mandrell R. E. Kim J. J. John C. M. Gibson B. W. Sugai J. V. Apicella M. A.. (1991). Endogenous sialylation of the lipooligosaccharides of *Neisseria meningitidis*. J. Bacteriol. 173, 2823–2832. doi: 10.1128/jb.173.9.2823-2832.1991, PMID: 1708379 PMC207863

[ref84] Mandrell R. E. Lesse A. J. Sugai J. V. Shero M. Griffiss J. M. Cole J. A.. (1990). *In vitro* and in vivo modification of *Neisseria gonorrhoeae* lipooligosaccharide epitope structure by sialylation. J. Exp. Med. 171, 1649–1664. doi: 10.1084/jem.171.5.1649, PMID: 1692081 PMC2187906

[ref85] Mason D. (1992). Autoimmunity. Sci. Prog. 76, 125–138. 1285283

[ref86] Masson L. Holbein B. E. Ashton F. E. (1982). Virulence linked to polysaccharide production in serogroup B *Neisseria meningitidis*. FEMS Microbiol. Lett. 13, 187–190. doi: 10.1111/j.1574-6968.1982.tb08253.x

[ref87] Meng X. Boons G.-J. Wösten M. M. S. M. Wennekes T. (2021). Metabolic labeling of legionaminic acid in flagellin glycosylation of *campylobacter jejuni* identifies Maf4 as a putative legionaminyl transferase. Angew. Chem. Int. Ed. Engl. 60, 24811–24816. doi: 10.1002/anie.202107181, PMID: 34519150 PMC9298399

[ref88] Michon F. Beurret M. Gamian A. Brisson J. R. Jennings H. J. (1990). Structure of the L5 lipopolysaccharide core oligosaccharides of *Neisseria meningitidis*. J. Biol. Chem. 265, 7243–7247. doi: 10.1016/S0021-9258(19)39105-7, PMID: 2110162

[ref89] Mielnik G. Gamian A. Doroszkiewicz W. (2001). Bactericidal activity of normal cord serum (NCS) against gram-negative rods with sialic acid-containing lipopolysaccharides (LPS). FEMS Immunol. Med. Microbiol. 31, 169–173. doi: 10.1111/j.1574-695X.2001.tb00516.x, PMID: 11720811

[ref90] Miller R. S. Hoskins L. C. (1981). Mucin degradation in human colon ecosystems. Fecal population densities of mucin-degrading bacteria estimated by a “most probable number” method. Gastroenterology 81, 759–765. doi: 10.1016/0016-5085(81)90503-5 7262520

[ref91] Mishu B. Blaser M. J. (1993). Role of infection due to *campylobacter jejuni* in the initiation of Guillain-Barré syndrome. Clin. Infect. Dis. Off. Publ. Infect. Dis. Soc. Am. 17, 104–108. doi: 10.1093/clinids/17.1.104, PMID: 8353228

[ref92] Moncla B. J. Braham P. Hillier S. L. (1990). Sialidase (neuraminidase) activity among gram-negative anaerobic and capnophilic bacteria. J. Clin. Microbiol. 28, 422–425. doi: 10.1128/jcm.28.3.422-425.1990, PMID: 2108991 PMC269635

[ref93] Moons S. J. Rossing E. Heming J. J. A. Janssen M. A. C. H. van Scherpenzeel M. Lefeber D. J.. (2021). Structure-activity relationship of fluorinated sialic acid inhibitors for bacterial sialylation. Bioconjug. Chem. 32, 1047–1051. doi: 10.1021/acs.bioconjchem.1c00194, PMID: 34043338 PMC8382218

[ref94] Moran A. P. Brandenburg K. Gutsmann T. Heine H. Holst O. Riekenberg S. (2009). Microbial glycobiology: Structures, relevance and applications. Amsterdam: Academic Press.

[ref95] Mortensen N. P. Kuijf M. L. Ang C. W. Schiellerup P. Krogfelt K. A. Jacobs B. C.. (2009). Sialylation of *campylobacter jejuni* lipooligosaccharides is associated with severe gastro-enteritis and reactive arthritis. Microbes Infect. 11, 988–994. doi: 10.1016/j.micinf.2009.07.004, PMID: 19631279

[ref96] Moustafa I. Connaris H. Taylor M. Zaitsev V. Wilson J. C. Kiefel M. J.. (2004). Sialic acid recognition by *vibrio cholerae* neuraminidase. J. Biol. Chem. 279, 40819–40826. doi: 10.1074/jbc.M404965200, PMID: 15226294

[ref97] Mukherjee K. Khatua B. Mandal C. (2020). Sialic acid-siglec-e interactions during *Pseudomonas aeruginosa* infection of macrophages interferes with phagosome maturation by altering intracellular calcium concentrations. Front. Immunol. 11:332. doi: 10.3389/fimmu.2020.00332, PMID: 32184783 PMC7059019

[ref98] Nachamkin I. Ung H. Moran A. P. Yoo D. Prendergast M. M. Nicholson M. A.. (1999). Ganglioside GM1 mimicry in *campylobacter* strains from sporadic infections in the United States. J Infect Dis 179, 1183–1189. doi: 10.1086/314725, PMID: 10191221

[ref99] Nicholson A. Lepow I. H. (1979). Host defense against *Neisseria meningitidis* requires a complement-dependent bactericidal activity. Science 205, 298–299. doi: 10.1126/science.451601, PMID: 451601

[ref100] Pawlak A. Rybka J. Dudek B. Krzyżewska E. Rybka W. Kędziora A.. (2017). Salmonella O48 serum resistance is connected with the elongation of the lipopolysaccharide O-antigen containing sialic acid. Int. J. Mol. Sci. 18:2022. doi: 10.3390/ijms18102022, PMID: 28934165 PMC5666704

[ref101] Penner J. L. Aspinall G. O. (1997). Diversity of lipopolysaccharide structures in campylobacter jejuni. J Infect Dis 176, S135–S138. doi: 10.1086/513778, PMID: 9396697

[ref102] Post D. M. B. Mungur R. Gibson B. W. MunsonJr R. S. (2005). Identification of a novel sialic acid transporter in *Haemophilus ducreyi*. Infect. Immun. 73, 6727–6735. doi: 10.1128/IAI.73.10.6727-6735.2005, PMID: 16177350 PMC1230923

[ref103] Pridmore A. C. Jarvis G. A. John C. M. Jack D. L. Dower S. K. Read R. C. (2003). Activation of toll-like receptor 2 (TLR2) and TLR4/MD2 by *Neisseria* is independent of capsule and lipooligosaccharide (LOS) sialylation but varies widely among LOS from different strains. Infect. Immun. 71, 3901–3908. doi: 10.1128/IAI.71.7.3901-3908.2003, PMID: 12819075 PMC161978

[ref104] Pridmore A. C. Wyllie D. H. Abdillahi F. Steeghs L. van der Ley P. Dower S. K.. (2001). A lipopolysaccharide-deficient mutant of *Neisseria meningitidis* elicits attenuated cytokine release by human macrophages and signals via toll-like receptor (TLR) 2 but not via TLR4/MD2. J Infect Dis 183, 89–96. doi: 10.1086/317647, PMID: 11076707

[ref105] Raetz C. R. Whitfield C. (2002). Lipopolysaccharide endotoxins. Annu. Rev. Biochem. 71, 635–700. doi: 10.1146/annurev.biochem.71.110601.135414, PMID: 12045108 PMC2569852

[ref106] Ram S. Sharma A. K. Simpson S. D. Gulati S. McQuillen D. P. Pangburn M. K.. (1998). A novel sialic acid binding site on factor H mediates serum resistance of sialylated Neisseria gonorrhoeae. J. Exp. Med. 187, 743–752. doi: 10.1084/jem.187.5.743, PMID: 9480984 PMC2212180

[ref107] Ramachandran G. (2014). Gram-positive and gram-negative bacterial toxins in sepsis: a brief review. Virulence 5, 213–218. doi: 10.4161/viru.27024, PMID: 24193365 PMC3916377

[ref108] Rhee S. H. (2014). Lipopolysaccharide: basic biochemistry, intracellular signaling, and physiological impacts in the gut. Intest. Res. 12, 90–95. doi: 10.5217/ir.2014.12.2.90, PMID: 25349574 PMC4204704

[ref109] Ringenberg M. A. Steenbergen S. M. Vimr E. R. (2003). The first committed step in the biosynthesis of sialic acid by *Escherichia coli* K1 does not involve a phosphorylated N-acetylmannosamine intermediate. Mol. Microbiol. 50, 961–975. doi: 10.1046/j.1365-2958.2003.03741.x, PMID: 14617154

[ref110] Robinson L. S. Lewis W. G. Lewis A. L. (2017). The sialate *O* -acetylesterase EstA from gut *Bacteroidetes* species enables sialidase-mediated cross-species foraging of 9- *O* -acetylated sialoglycans. J. Biol. Chem. 292, 11861–11872. doi: 10.1074/jbc.M116.769232, PMID: 28526748 PMC5512079

[ref111] Roy S. Douglas C. W. I. Stafford G. P. (2010). A novel sialic acid utilization and uptake system in the periodontal pathogen Tannerella forsythia. J. Bacteriol. 192, 2285–2293. doi: 10.1128/JB.00079-10, PMID: 20190043 PMC2863479

[ref112] Sandler R. D. Hoggard N. Hadjivassiliou M. (2015). Miller-fisher syndrome: is the ataxia central or peripheral? Cereb. Ataxias 2:3. doi: 10.1186/s40673-015-0021-3, PMID: 26331046 PMC4552373

[ref113] Sato C. Kitajima K. (2021). Polysialylation and disease. Mol. Aspects Med. 79:100892. doi: 10.1016/j.mam.2020.100892, PMID: 32863045

[ref114] Schauer R. (2009). Sialic acids as regulators of molecular and cellular interactions. Curr. Opin. Struct. Biol. 19, 507–514. doi: 10.1016/j.sbi.2009.06.003, PMID: 19699080 PMC7127376

[ref115] Schauer R. Kamerling J. P. (2018). Exploration of the sialic acid world. Adv. Carbohydr. Chem. Biochem. 75, 1–213. doi: 10.1016/bs.accb.2018.09.001, PMID: 30509400 PMC7112061

[ref116] Schweda E. K. Jonasson J. A. Jansson P. E. (1995). Structural studies of lipooligosaccharides from Haemophilus ducreyi ITM 5535, ITM 3147, and a fresh clinical isolate, ACY1: evidence for intrastrain heterogeneity with the production of mutually exclusive sialylated or elongated glycoforms. J. Bacteriol. 177, 5316–5321. doi: 10.1128/jb.177.18.5316-5321.1995, PMID: 7665520 PMC177325

[ref117] Sela D. A. Chapman J. Adeuya A. Kim J. H. Chen F. Whitehead T. R.. (2008). The genome sequence of Bifidobacterium longum subsp. infantis reveals adaptations for milk utilization within the infant microbiome. Proc. Natl. Acad. Sci. 105, 18964–18969. doi: 10.1073/pnas.0809584105, PMID: 19033196 PMC2596198

[ref118] Severi E. Hood D. W. Thomas G. H. (2007). Sialic acid utilization by bacterial pathogens. Microbiol. Read. Engl. 153, 2817–2822. doi: 10.1099/mic.0.2007/009480-0, PMID: 17768226

[ref119] Severi E. Randle G. Kivlin P. Whitfield K. Young R. Moxon R.. (2005). Sialic acid transport in *Haemophilus influenzae* is essential for lipopolysaccharide sialylation and serum resistance and is dependent on a novel tripartite ATP-independent periplasmic transporter: sialic acid TRAP transporter in *Haemophilus influenzae*. Mol. Microbiol. 58, 1173–1185. doi: 10.1111/j.1365-2958.2005.04901.x, PMID: 16262798

[ref120] Sheikhi Moghaddam L. Adegbite A. McCarthy P. C. (2021). Investigation of bioluminescence-based assays for determination of kinetic parameters for the bifunctional Neisseria meningitidis serogroup W capsule polymerase. BMC. Res. Notes 14:417. doi: 10.1186/s13104-021-05831-1, PMID: 34794506 PMC8600345

[ref121] Shell D. M. Chiles L. Judd R. C. Seal S. Rest R. F. (2002). The *Neisseria* lipooligosaccharide-specific alpha-2,3-sialyltransferase is a surface-exposed outer membrane protein. Infect. Immun. 70, 3744–3751. doi: 10.1128/IAI.70.7.3744-3751.2002, PMID: 12065517 PMC128106

[ref122] Shen G.-J. Datta A. K. Izumi M. Koeller K. M. Wong C.-H. (1999). Expression of α2,8/2,9-Polysialyltransferase from *Escherichia coli* K92: characterization of the enzyme and its reaction products. J. Biol. Chem. 274, 35139–35146. doi: 10.1074/jbc.274.49.35139, PMID: 10574996

[ref123] Soong G. (2006). Bacterial neuraminidase facilitates mucosal infection by participating in biofilm production. J. Clin. Invest. 116, 2297–2305. doi: 10.1172/JCI27920, PMID: 16862214 PMC1513050

[ref124] Spinola S. M. Li W. Fortney K. R. Janowicz D. M. Zwickl B. Katz B. P.. (2012). Sialylation of lipooligosaccharides is dispensable for the virulence of *Haemophilus ducreyi* in humans. Infect. Immun. 80, 679–687. doi: 10.1128/IAI.05826-11, PMID: 22144477 PMC3264291

[ref125] Spitalnik S. L. Schwartz J. F. Magnani J. L. Roberts D. D. Spitalnik P. F. Civin C. I.. (1985). Anti-My-28, an antigranulocyte mouse monoclonal antibody, binds to a sugar sequence in lacto-N-neotetraose. Blood 66, 319–326. doi: 10.1182/blood.V66.2.319.319, PMID: 3926023

[ref126] Steenbergen S. M. Lee Y.-C. Vann W. F. Vionnet J. Wright L. F. Vimr E. R. (2006). Separate pathways for o acetylation of polymeric and monomeric sialic acids and identification of Sialyl O-acetyl esterase in *Escherichia coli* K1. J. Bacteriol. 188, 6195–6206. doi: 10.1128/JB.00466-06, PMID: 16923886 PMC1595355

[ref127] Steenbergen S. M. Lichtensteiger C. A. Caughlan R. Garfinkle J. Fuller T. E. Vimr E. R. (2005). Sialic acid metabolism and systemic pasteurellosis. Infect. Immun. 73, 1284–1294. doi: 10.1128/IAI.73.3.1284-1294.2005, PMID: 15731025 PMC1064920

[ref128] Thomas G. H. (2016). Sialic acid acquisition in bacteria-one substrate, many transporters. Biochem. Soc. Trans. 44, 760–765. doi: 10.1042/BST20160056, PMID: 27284039

[ref129] Tiralongo J. (2010). “Sialic acid-specific microbial lectins,” in Microbial Glycobiology. eds. Holst O. Brennan P. J. Itzstein M. V. Moran A. P. (San Diego, CA: Academic Press). 585–598.

[ref130] Trappetti C. Kadioglu A. Carter M. Hayre J. Iannelli F. Pozzi G.. (2009). Sialic acid: a preventable signal for pneumococcal biofilm formation, colonization, and invasion of the host. J Infect Dis 199, 1497–1505. doi: 10.1086/598483, PMID: 19392624

[ref131] Traving C. Schauer R. (1998). Structure, function and metabolism of sialic acids. Cell. Mol. Life Sci. CMLS 54, 1330–1349. doi: 10.1007/s000180050258, PMID: 9893709 PMC7082800

[ref132] Unkmeir A. Kämmerer U. Stade A. Hübner C. Haller S. Kolb-Mäurer A.. (2002). Lipooligosaccharide and polysaccharide capsule: virulence factors of *Neisseria meningitidis* that determine meningococcal interaction with human dendritic cells. Infect. Immun. 70, 2454–2462. doi: 10.1128/IAI.70.5.2454-2462.2002, PMID: 11953382 PMC127941

[ref133] Van Calsteren M.-R. Gagnon F. Calzas C. Goyette-Desjardins G. Okura M. Takamatsu D.. (2013). Structure determination of *Streptococcus suis* serotype 14 capsular polysaccharide. Biochem. Cell Biol. 91, 49–58. doi: 10.1139/bcb-2012-0036, PMID: 23527632

[ref134] van der Woude M. W. Baumler A. J. (2004). Phase and antigenic variation in bacteria. Clin. Microbiol. Rev. 17, 581–611. doi: 10.1128/CMR.17.3.581-611.2004, PMID: 15258095 PMC452554

[ref135] Varki A. (1992). Diversity in the sialic acids. Glycobiology 2, 25–40. doi: 10.1093/glycob/2.1.25, PMID: 1550987 PMC7108601

[ref136] Varki A. (2008). Sialic acids in human health and disease. Trends Mol. Med. 14, 351–360. doi: 10.1016/j.molmed.2008.06.002, PMID: 18606570 PMC2553044

[ref137] Varki A. (2017). Are humans prone to autoimmunity? Implications from evolutionary changes in hominin sialic acid biology. J. Autoimmun. 83, 134–142. doi: 10.1016/j.jaut.2017.07.011, PMID: 28755952

[ref138] Varki A. Schauer R. (2009). “Sialic Acids,” in Essentials of Glycobiology. eds. Varki A. Cummings R. D. Esko J. D. Freeze H. H. Stanley P. Bertozzi C. R.. (Cold Spring Harbor (NY): Cold Spring Harbor Laboratory Press). 20301239

[ref139] Vaure C. Liu Y. (2014). A comparative review of toll-like receptor 4 expression and functionality in different animal species. Front. Immunol. 5:316. doi: 10.3389/fimmu.2014.00316, PMID: 25071777 PMC4090903

[ref140] Vimr E. R. (2013). Unified theory of bacterial sialometabolism: how and why bacteria metabolize host sialic acids. ISRN Microbiol. 2013, 1–26. doi: 10.1155/2013/816713, PMID: 23724337 PMC3658417

[ref141] Vimr E. R. Kalivoda K. A. Deszo E. L. Steenbergen S. M. (2004). Diversity of microbial sialic acid metabolism. Microbiol. Mol. Biol. Rev. 68, 132–153. doi: 10.1128/MMBR.68.1.132-153.2004, PMID: 15007099 PMC362108

[ref142] Vimr E. Lichtensteiger C. Steenbergen S. (2000). Sialic acid metabolism’s dual function in Haemophilus influenzae. Mol. Microbiol. 36, 1113–1123. doi: 10.1046/j.1365-2958.2000.01925.x, PMID: 10844695

[ref143] Vimr E. R. Troy F. A. (1985). Identification of an inducible catabolic system for sialic acids (nan) in Escherichia coli. J. Bacteriol. 164, 845–853. doi: 10.1128/jb.164.2.845-853.1985, PMID: 3902799 PMC214328

[ref144] Vinogradov E. Michael F. Homma K. Sharma A. Cox A. D. (2017). Structure of the LPS O-chain from *fusobacterium nucleatum* strain 10953, containing sialic acid. Carbohydr. Res. 440-441, 38–42. doi: 10.1016/j.carres.2017.01.009, PMID: 28199859 PMC5502818

[ref145] Vogel U. Weinberger A. Frank R. Müller A. Köhl J. Atkinson J. P.. (1997). Complement factor C3 deposition and serum resistance in isogenic capsule and lipooligosaccharide sialic acid mutants of serogroup B *Neisseria meningitidis*. Infect. Immun. 65, 4022–4029. doi: 10.1128/iai.65.10.4022-4029.1997, PMID: 9317002 PMC175578

[ref146] Wetzler L. M. Barry K. Blake M. S. Gotschlich E. C. (1992). Gonococcal lipooligosaccharide sialylation prevents complement-dependent killing by immune sera. Infect. Immun. 60, 39–43. doi: 10.1128/iai.60.1.39-43.1992, PMID: 1729195 PMC257500

[ref147] Wong A. Grau M. A. Singh A. K. Woodiga S. A. King S. J. (2018). Role of neuraminidase-producing bacteria in exposing cryptic carbohydrate receptors for *Streptococcus gordonii* adherence. Infect. Immun. 86, e00068–e00018. doi: 10.1128/IAI.00068-18, PMID: 29661931 PMC6013669

[ref148] Xu X. Tong T. Yang X. Pan Y. Lin L. Li C. (2017). Differences in survival, virulence and biofilm formation between sialidase-deficient and W83 wild-type *Porphyromonas gingivalis* strains under stressful environmental conditions. BMC Microbiol. 17:178. doi: 10.1186/s12866-017-1087-2, PMID: 28821225 PMC5563019

[ref149] Yamashita K. Tachibana Y. Kobata A. (1976). Oligosaccharides of human milk. Arch. Biochem. Biophys. 174, 582–591. doi: 10.1016/0003-9861(76)90387-8 1230009

[ref150] Yang J. Ma W. Wu Y. Zhou H. Song S. Cao Y.. (2021). O-acetylation of capsular polysialic acid enables *Escherichia coli* K1 escaping from Siglec-mediated innate immunity and lysosomal degradation of *E. coli*-containing vacuoles in macrophage-like cells. Microbiol. Spectr. 9. doi: 10.1128/spectrum.00399-21, PMID: 34878295 PMC8653822

[ref151] Yang X. Pan Y. Xu X. Tong T. Yu S. Zhao Y.. (2018). Sialidase deficiency in *Porphyromonas gingivalis* increases IL-12 secretion in stimulated macrophages through regulation of CR3, IncRNA GAS5 and miR-21. Front. Cell. Infect. Microbiol. 8:100. doi: 10.3389/fcimb.2018.00100, PMID: 29675399 PMC5895773

[ref152] Yuki N. Takahashi M. Tagawa Y. Kashiwase K. Tadokoro K. Sairo K. (1997). Association of *Campylobacter jejuni* serotype with antiganglioside antibody in Guillain-Barré syndrome and Fisher’s syndrome: association of *Campylobacter* serotype with antiganglioside antibody. Ann. Neurol. 42, 28–33. doi: 10.1002/ana.410420107, PMID: 9225682

[ref153] Yuki N. Taki T. Takahashi M. Saito K. Yoshino H. Tai T.. (1994). Molecular mimicry between GQ1b ganglioside and lipopolysaccharides of *campylobacter jejuni* isolated from patients with Fisher’s syndrome. Ann. Neurol. 36, 791–793. doi: 10.1002/ana.410360517, PMID: 7526777

